# Genomic autopsy to identify underlying causes of pregnancy loss and perinatal death

**DOI:** 10.1038/s41591-022-02142-1

**Published:** 2023-01-19

**Authors:** Alicia B. Byrne, Peer Arts, Thuong T. Ha, Karin S. Kassahn, Lynn S. Pais, Anne O’Donnell-Luria, Broad Institute Center for Mendelian Genomics, François Aguet, Harindra M. Arachchi, Christina A. Austin-Tse, Larry Babb, Samantha M. Baxter, Harrison Brand, Jaime Chang, Katherine R. Chao, Ryan L. Collins, Beryl Cummings, Kayla Delano, Stephanie P. DiTroia, Eleina England, Emily Evangelista, Selin Everett, Laurent C. Francioli, Jack Fu, Vijay S. Ganesh, Kiran V. Garimella, Laura D. Gauthier, Julia K. Goodrich, Sanna Gudmundsson, Stacey J. Hall, Yongqing Huang, Steve Jahl, Kristen M. Laricchia, Kathryn E. Larkin, Monkol Lek, Gabrielle Lemire, Rachel B. Lipson, Alysia Kern Lovgren, Daniel G. MacArthur, Brian E. Mangilog, Stacy Mano, Jamie L. Marshall, Thomas E. Mullen, Kevin K. Nguyen, Emily O’Heir, Melanie C. O’Leary, Ikeoluwa A. Osei-Owusu, Jorge Perez de Acha Chavez, Emma Pierce-Hoffman, Heidi L. Rehm, Jillian Serrano, Moriel Singer-Berk, Hana Snow, Matthew Solomonson, Rachel G. Son, Abigail Sveden, Michael Talkowski, Grace Tiao, Miriam S. Udler, Zaheer Valivullah, Elise Valkanas, Grace E. VanNoy, Qingbo S. Wang, Nicholas A. Watts, Ben Weisburd, Clara E. Williamson, Michael W. Wilson, Lauren Witzgall, Monica H. Wojcik, Isaac Wong, Jordan C. Wood, Shifa Zhang, Milena Babic, Mahalia S. B. Frank, Jinghua Feng, Paul Wang, David M. Lawrence, Leila Eshraghi, Luis Arriola, John Toubia, Hung Nguyen, Genomic Autopsy Study Research Network, Disna Abeysuriya, Lesley C. Ades, David J. Amor, Susan Arbuckle, Madhura Bakshi, Bligh Berry, Tiffany Boughtwood, Adam Bournazos, Alessandra Bray, Fiona Chan, Yuen Chan, Clara Chung, Jonathan Clark, Jackie Collett, Alison Colley, Felicity Collins, Sandra Cooper, Mark A. Corbett, Jane E. Dahlstrom, Peter Dargaville, Janene Davies, Tenielle Davis, Jarrad Dearman, Jayanthi Dissanayake, Julia Dobbins, Helen Doyle, Andrew Dubowsky, Matt Edwards, Lisa J. Ewans, Mitali Fadia, Andrew Fennell, Keri Finlay, Andrew French, Kathryn Friend, Alison E. Gardner, Jozef Gecz, Nicole Graf, Eric A. Haan, Georgina Hollingsworth, Ari E. Horton, Denise Howting, Matthew F. Hunter, Gareth Jevon, Benjamin Kamien, Debra Kennedy, T. Yee Khong, Michael Krivanek, Thessa Kroes, Emma I. Krzesinski, Edward Kwan, Stephanie Lau, Shannon LeBlanc, Jan Liebelt, Suzanna Lindsey-Temple, Jill Lipsett, Christine K. C. Loo, Julia Low, Amali Mallawaarachchi, Nick Manton, Admire Matsika, Tessa Mattiske, Julie McGaughran, Lesley McGregor, Namita Mittal, Ali Moghimi, Lynette Moore, Hatice Mutlu Albayrak, Jessica Ng, Jillian Nicholl, Nicholas Pachter, John Papadimitriou, Renae Parker, Sarah Parsons, Chirag Patel, Rhonda Pawlowski, Luis A. Perez-Jurado, Jason R. Pinner, Katerina Politis, Cathryn Poulton, Theresa Power, Michael Quinn, Sulekha Rajagopalan, Matthew Regan, Jonathan Rodgers, Steuart Rorke, Rani Sachdev, Suzanne Sallevelt, Sarah A. Sandaradura, Maryam Shamassi, Roshan Shamon, Isabella Sherburn, Jennie Slee, Annalisa Solinas, Ella Sugo, Elizabeth Thompson, Sagarika Tripathy, Anand Vasudevan, Melisa Vazquez, Kunal Verma, Mthulisi Viki, Mathew Wallis, Dani L. Webber, Martin Weber, Karen Whale, Meredith Wilson, Lisa Worgan, Sui Yu, George McGillivray, Jason Pinner, Fiona McKenzie, Rebecca Morrow, Jill Lipsett, Nick Manton, T. Yee Khong, Lynette Moore, Jan E. Liebelt, Andreas W. Schreiber, Sarah L. King-Smith, Tristan S. E. Hardy, Matilda R. Jackson, Christopher P. Barnett, Hamish S. Scott

**Affiliations:** 1 Department of Genetics and Molecular Pathology, Centre for Cancer Biology, an alliance between SA Pathology and the University of South Australia, Adelaide, South Australia Australia; 2 UniSA Clinical and Health Sciences, University of South Australia, Adelaide, South Australia Australia; 3 Broad Institute of MIT and Harvard, Cambridge, MA USA; 4 Adelaide Medical School, University of Adelaide, Adelaide, South Australia Australia; 5 ACRF Genomics Facility, Centre for Cancer Biology, an alliance between SA Pathology and the University of South Australia, Adelaide, South Australia Australia; 6 Department of Genetics and Molecular Pathology, SA Pathology, Adelaide, South Australia Australia; 7 Division of Genetics and Genomics, Boston Childrens Hospital, Boston, MA USA; 8 Victorian Clinical Genetics Services, Murdoch Children’s Research Institute and Royal Women’s Hospital, Melbourne, Victoria Australia; 9 Centre for Clinical Genetics, Sydney Children’s Hospitals Network – Randwick, Sydney, New South Wales Australia; 10 School of Women’s and Children’s Health, University of NSW, Sydney, New South Wales Australia; 11 Genetic Services of Western Australia, Perth, Western Australia Australia; 12 School of Paediatrics and Child Health, University of Western Australia, Perth, Western Australia Australia; 13 Department of Anatomical Pathology, SA Pathology, Women’s and Children’s Hospital, North Adelaide, South Australia Australia; 14 Paediatric and Reproductive Genetics Unit, South Australian Clinical Genetics Service, Women’s and Children’s Hospital, North Adelaide, South Australia Australia; 15 Repromed, Dulwich, South Australia Australia; 16 School of Biological Sciences, University of Adelaide, Adelaide, South Australia Australia; 17 Australian Genomics, Parkville, Victoria Australia; 18 Center for Genomic Medicine, Massachusetts General Hospital, Boston, MA USA; 19 Program in Bioinformatics and Integrative Genomics, Harvard Medical School, Boston, MA USA; 20 Neuromuscular Division, Department of Neurology, Brigham and Women’s Hospital, Boston, MA USA; 21 Yale School of Medicine, Department of Genetics, New Haven, CT USA; 22 Garvan Institute of Medical Research and UNSW Sydney, Centre for Population Genomics, Sydney, New South Wales Australia; 23 Murdoch Children’s Research Institute, Centre for Population Genomics, Melbourne, Victoria Australia; 24 Boston Childrens Hospital Divisions of Newborn Medicine and Genetics and Genomics, Boston, MA USA; 25 Department of Anatomical Pathology, Perth Children’s Hospital, Nedlands, Western Australia Australia; 26 Department of Clinical Genetics, Children’s Hospital at Westmead, Westmead, New South Wales Australia; 27 Disciplines of Child and Adolescent Health and Genomic Medicine, University of Sydney, Sydney, New South Wales Australia; 28 Murdoch Children’s Research Institute, Parkville, Victoria Australia; 29 Department of Histopathology, The Children’s Hospital at Westmead, Westmead, New South Wales Australia; 30 Department of Clinical Genetics, Liverpool Hospital, Liverpool, New South Wales Australia; 31 Kids Neuroscience Centre, The Children’s Hospital at Westmead, Westmead, New South Wales Australia; 32 Faculty of Medicine and Health, The University of Sydney, Westmead, New South Wales Australia; 33 Department of Anatomical Pathology, Royal Children’s Hospital, Parkville, Victoria Australia; 34 Department of Pathology, Monash Health, Melbourne, Victoria Australia; 35 Department of Anatomical Pathology, Austin Health, Melbourne, Victoria Australia; 36 Clinical Genetics Service, Institute of Precision Medicine and Bioinformatics, Royal Prince Alfred Hospital, Sydney, New South Wales Australia; 37 Functional Neuromics, Children’s Medical Research Institute, Westmead, New South Wales Australia; 38 Robinson Research Institute, The University of Adelaide, Adelaide, South Australia Australia; 39 Department of Anatomical Pathology, Canberra Hospital, Canberra, Australian Capital Territory Australia; 40 ANU Medical School, Canberra, Australian Capital Territory Australia; 41 Neonatal and Pediatric Intensive Care Unit, Department of Pediatrics, Royal Hobart Hospital, Hobart, Tasmania Australia; 42 Department of Pathology, Royal Brisbane and Women’s Hospital, Herston, Brisbane, Queensland Australia; 43 Royal Women’s Hospital, Melbourne, Victoria Australia; 44 Department of Anatomical Pathology, Dorevitch Pathology, Laboratory, Frankston Hospital, Peninsula Health, Victoria Australia; 45 Hunter Genetics, Newcastle, New South Wales Australia; 46 St Vincent’s Clinical School, University of NSW, Sydney, New South Wales Australia; 47 Kinghorn Centre for Clinical Genomics, Garvan Institute of Medical Research, Sydney, New South Wales Australia; 48 Monash Genetics, Monash Health, Melbourne, Victoria Australia; 49 Department of Pediatrics, Monash University, Melbourne, Victoria Australia; 50 Genetics and Molecular Pathology, SA Pathology at Women’s and Children’s Hospital, North Adelaide, South Australia Australia; 51 MotherSafe, Royal Hospital for Women, Sydney, New South Wales Australia; 52 Department of Anatomical Pathology, SA Pathology, Women’s and Children’s Hospital, North Adelaide, South Australia Australia; 53 Department of Anatomical Pathology, NSW Health Pathology, Prince of Wales Hospital, Randwick, New South Wales Australia; 54 Department of Medical Genomics, Royal Prince Alfred Hospital, Sydney, New South Wales Australia; 55 Department of Anatomical Pathology, Mater Health Services, Brisbane, Queensland Australia; 56 Genetic Health Queensland, Royal Brisbane and Women’s Hospital, Brisbane, Queensland Australia; 57 Department of Pediatric Genetics, Ankara Bilkent City Hospital Children’s Hospital, Ankara, Turkey; 58 Victorian Institute of Forensic Medicine, Southbank, Victoria Australia; 59 Genetics Service, Hospital del Mar Medical Research Institute, Network Research Centre for Rare Diseases, Barcelona, Spain; 60 Department of Medicine and Life Sciences, Universitat Pompeu Fabra, Barcelona, Spain; 61 Royal Prince Alfred Hospital, Camperdown, New South Wales Australia; 62 Department of Anatomical Pathology, Pathology North Hunter, New Lambton, New South Wales Australia; 63 Centre for Translational Pathology, University of Melbourne, Parkville, Victoria Australia; 64 Genomic Medicine, The Royal Melbourne Hospital, Parkville, Victoria Australia; 65 School of Medicine and Menzies Institute for Medical Research, University of Tasmania, Hobart, Tasmania Australia; 66 Department of Pathology, The Royal Hobart Hospital, Hobart, Tasmania Australia

**Keywords:** Genetic testing, Disease genetics, Medical genomics

## Abstract

Pregnancy loss and perinatal death are devastating events for families. We assessed ‘genomic autopsy’ as an adjunct to standard autopsy for 200 families who had experienced fetal or newborn death, providing a definitive or candidate genetic diagnosis in 105 families. Our cohort provides evidence of severe atypical in utero presentations of known genetic disorders and identifies novel phenotypes and disease genes. Inheritance of 42% of definitive diagnoses were either autosomal recessive (30.8%), X-linked recessive (3.8%) or autosomal dominant (excluding de novos, 7.7%), with risk of recurrence in future pregnancies. We report that at least ten families (5%) used their diagnosis for preimplantation (5) or prenatal diagnosis (5) of 12 pregnancies. We emphasize the clinical importance of genomic investigations of pregnancy loss and perinatal death, with short turnaround times for diagnostic reporting and followed by systematic research follow-up investigations. This approach has the potential to enable accurate counseling for future pregnancies.

## Abstract (web summary)

In a new study including 200 families who experienced perinatal death, adding genomic analyses to standard autopsies improved the identification of underlying pathogenic causes and informed genetic counseling.

## Main

In developed countries, approximately 1% of pregnancies result in pregnancy loss, termination of pregnancy or perinatal death, which is the collective term for the loss of a fetus (stillbirths >20 weeks or >400 g) or neonate (up to 28 days post birth)^1–4^. Despite advanced monitoring of pregnancies and increased access to healthcare, eight fetal and neonatal deaths are experienced per day in Australia, a figure that has not changed over the past two decades^2^. The devastating impact of pregnancy loss, terminations and perinatal death on families and the wider community is often further compounded by the uncertainty of the cause of death and the subsequent recurrence risk for future pregnancies^5–7^. Clinical testing to determine the underlying etiological factors involved in the death currently involves the complex integration of family and obstetric history, radiographic imaging and macroscopic and histological examination of the body and placenta, along with laboratory investigations such as biochemistry, microbiology and genetic testing^8,9^. While collectively these investigations are most likely to yield a clinical diagnosis, for complex reasons—including the perceived invasiveness of perinatal autopsy and religious or cultural beliefs—an autopsy is performed in <50% of cases^1,4,10^. Congenital abnormalities are present in one-third of cases, either as the main determinant of fetal death in utero (for example, via hydrops fetalis) or, more commonly, as the main precedent for a termination of pregnancy. Even with congenital abnormalities, for the majority of cases an underlying etiology is not determined by current standard-of-care practices. A further 10.5% of deaths remain completely unexplained despite extensive investigation^1,11^.

A genetic etiology is expected to underpin fetal and neonatal loss due to congenital abnormality, along with many cases of unexplained death, often representing an extreme phenotype and one that is incompatible with life. Large chromosomal abnormalities, such as autosomal trisomies and copy number variants (CNVs), account for 25–30% of cases with congenital abnormalities and are routinely detected by microarray, performed as part of standard-of-care autopsy^11,12^. A further ~5% of cases are attributed to monogenic disorders, diagnosed in the clinical setting by single-gene or gene panel testing. However, these investigations are performed only if a specific phenotype is suspected^11^. As a result of the limited genetic investigation that is currently considered standard-of-care, the underlying etiology of ~70% of congenital abnormality-related deaths remains unexplained, limiting the accuracy of counseling and restricting options to prevent recurrence.

A broader approach to identification of the molecular origins of congenital abnormalities and unexplained perinatal death, by exome sequencing (ES) or genome sequencing (GS), would allow the full range of large-scale genetic variation discernible by microarray, as well as identification of single-nucleotide variants (SNVs) and insertions/deletions (indels)^13^.

In this study we investigated the utility of a genomic autopsy by offering ES or GS to families who had experienced pregnancy loss, including terminations or perinatal death due to congenital abnormality without a genetic diagnosis identified from standard-of-care testing or where death was unexplained. The primary objective was to provide families with diagnoses, accurate recurrence risks and options for prenatal diagnosis (PND) or preimplantation genetic diagnosis (PGD) in future pregnancies. Supporting this aim was the systematic search for additional kindreds, in conjunction with adjunct investigations such as RNA sequencing (RNA-seq) and in vitro and in vivo studies, to implicate causality of novel variants and genes. Here we report the results of our ongoing study on genomic autopsy and the subsequent clinical impact for the first 200 consecutively referred families. We demonstrate the importance of reducing turnaround times for mendeliome (known online Mendelian inheritance in man (OMIM)-morbid genes) analysis for perinatal death so that the genetic diagnosis can inform future pregnancies. Furthermore, we highlight the utility of experimental follow-up and identification of additional cases to establish a final diagnosis in this understudied, deceased patient cohort, with a high rate of variants in novel candidate genes and phenotype expansions.

## Results

### Cohort characteristics

**Extended Data Fig. 1:**
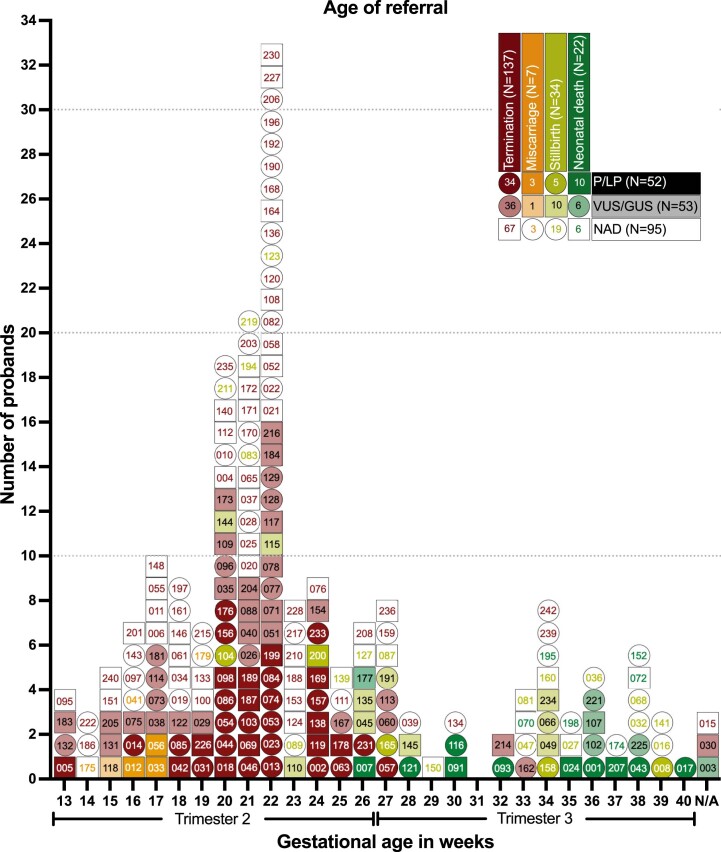
Cohort distribution of (gestational) age, sex and classification of death. Shows the distribution in (gestational) age, sex and death classification within the perinatal period of the 200 probands in our cohort. Only completed gestational weeks are displayed. Circle, female proband; square, male proband; red, termination of pregnancy; orange, miscarriage; light green, stillbirth; dark green, neonatal death; darker shading, LP/P variant; lighter shading, Variant or Gene of Uncertain Significance (VUS/GUS);, open box, no abnormality detected (NAD).

**Extended Data Fig. 2:**
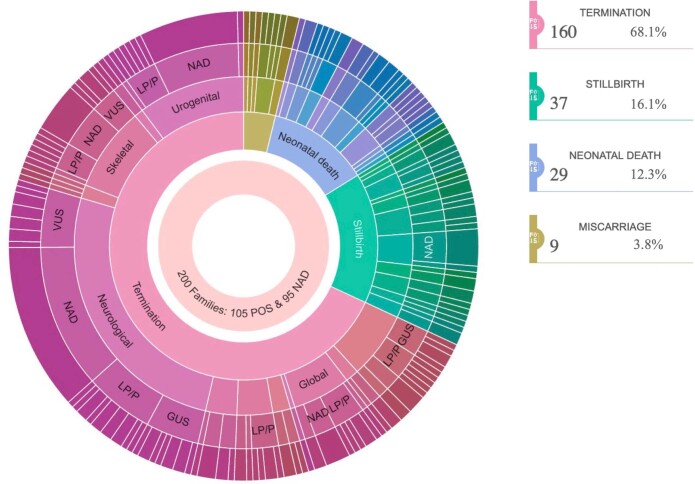
Sunburst diagram of the complete cohort. A sunburst plot representing all 200 families separated by the type of pregnancy loss (2nd, most inner ring), across different organ systems (third ring), the genomic findings (fourth ring) and candidate disease genes (fifth, most outer ring). The genomic findings are separated by LP/P ((Likely) pathogenic), VUS (variant of uncertain significance), GUS (gene of uncertain significance), and NAD (no abnormalities detected). The percentages in the right-side legend represent the number of variants identified across the different pregnancy groups. This plot shows that the study cohort mostly consisted of termination of pregnancies. The input data for the sunburst plot was generated from Supplementary Table 2. All sunburst plots were generated using the sunburstR package (https://github.com/timelyportfolio/sunburstR).

**Extended Data Fig. 3:**
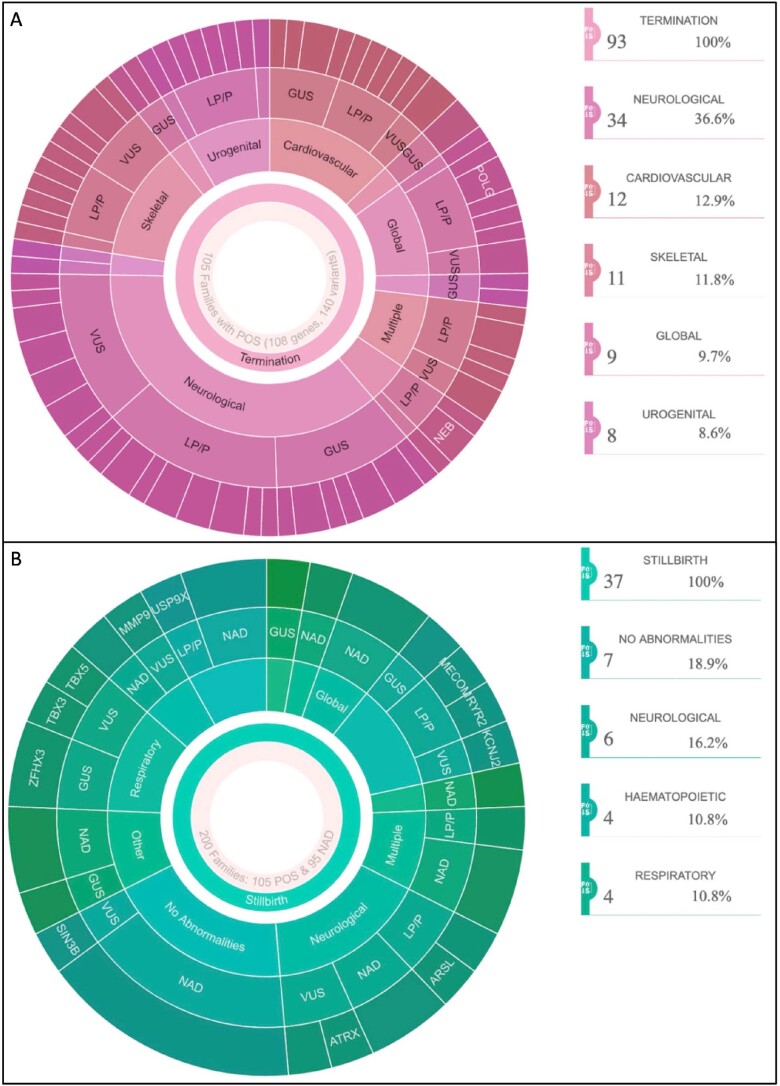
Sunburst diagrams showing the characteristics of the terminated pregnancies and stillbirths. (**a)** Sunburst plot representing the group of pregnancies terminated, due to in utero abnormalities, and the subsequent genomic findings. The five most affected organ systems within this subgroup are shown, with the neurological phenotypes yielding the highest number of diagnoses (36.6%). Multiple refers to two or more different organ systems affected. The genomic findings are separated by LP,P (Likely pathogenic, pathogenic), VUS (variant of uncertain significance), GUS (gene of uncertain significance), and NAD (no abnormalities detected). **(b)** Sunburst plot representing the group of pregnancies with stillbirth cases; pregnancy losses beyond 20 weeks of gestation. This plot shows that a majority of stillbirth cases with no genomic findings (NAD) are those with no congenital abnormalities (18.9%) identified, meanwhile stillbirth cases with affected respiratory or hematopoietic systems are more likely to yield a candidate disease gene; 7/7 POS (100%).

**Extended Data Fig. 4:**
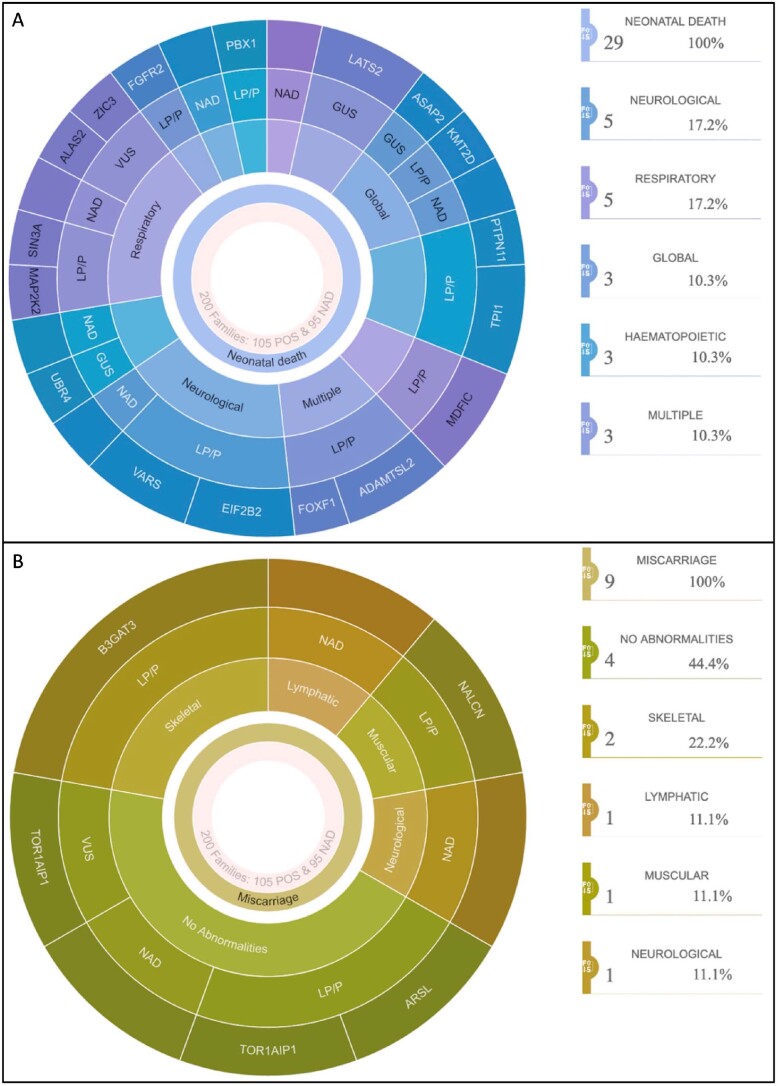
Sunburst diagrams showing the characteristics of the Neonatal deaths and miscarriages. **(a)** Sunburst plot representing families with neonatal death; loss of an infant up to 28 days old. This plot shows that out of the four subgroups of fetal and neonatal loss, cases with neonatal deaths have the highest number of genomic findings (23/29 POS and 6/29 NAD). Neonates with respiratory and neurological abnormalities represent one third of this subgroup. Neonates with two or more major organ systems affected, and hematopoietic abnormalities, have 100% genomic findings in known and established disease genes (ADAMTSL2, FOXF1, PTPN11 and TPI1 respectively). **(b)** Sunburst plot representing the smallest subgroup within the study cohort, miscarriages; pregnancy losses before 20 weeks of gestation. This plot shows that there is a 66.7% chance of discovering candidate disease genes (6/9 POS) from early pregnancy loss, with and without congenital abnormalities detected in utero.

The study cohort was selected based on families consecutively referred through the Genomic Autopsy Study clinical network, after exclusion of cases with a molecular diagnosis established from previous array or gene panel testing. The cohort demographics including sex, gestational age (Extended Data Fig. 1), reason for referral, clinical history (pregnancy, maternal and paternal) and major organ system affected; detailed phenotypes with Human Phenotype Ontology (HPO) terms are reported in Supplementary Tables 1 and 2. The largest proportion of cases (68.5%, 137 of 200) were terminations of pregnancy due to structural abnormalities observed on ultrasound. The remaining 63 cases were spontaneous deaths that occurred either in utero (*n* = 41) or in the neonatal period (*n* = 22) (Extended Data Figs. 1–4).

### Identification of disease-causing variants from mendeliome analysis

**Fig. 1:**
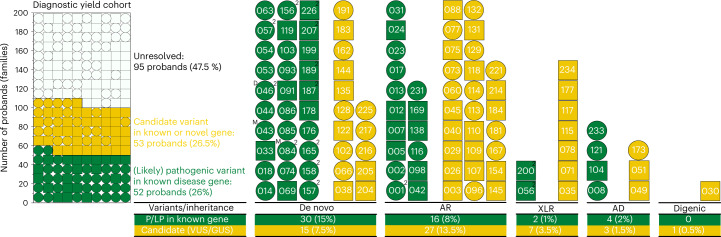
Diagnostic yield of pregnancy loss and perinatal death cohort with observed inheritance models. Corresponding pedigree number is contained within each proband symbol. AD, autosomal dominant; AR, autosomal recessive; GUS, gene of uncertain significance; LP, likely pathogenic; P, pathogenic; XLR, X-linked recessive; VUS, variant of uncertain significance; ^D^, dual diagnosis; ^M^, parental mosaic indicates recurrence risk >1%; ^2^, families with recurrently mutated genes; circles, female probands; squares, male probands; green, LP/P variant; yellow, candidate variant; white, unresolved.

**Extended Data Fig. 5:**
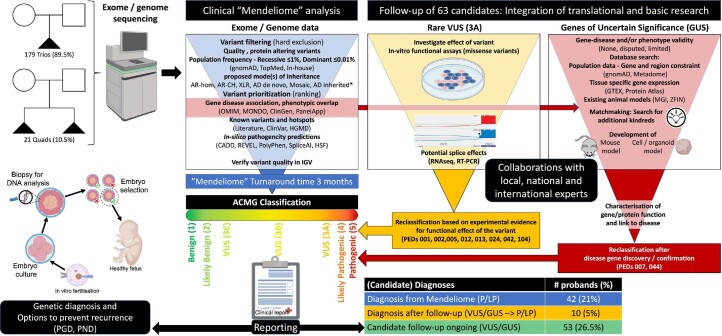
Workflow schematic of the first 200 families in the Genomic Autopsy study. Schematic diagram of the workflow and analysis of the Genomic Autopsy Study with the goal to provide answers to families and prevent recurrence of pregnancy loss and perinatal death. Highlighting the separate clinical-grade analysis and integrated research follow-up. Mendeliome, OMIM morbid genes; AR:,Autosomal Recessive; hom, homozygous; CH, Compound Heterozygous,;XLR, X-Linked Recessive; AD, Autosomal Dominant,;VUS, Variant of Uncertain Significance; GUS, Gene of Uncertain Significance; ACMG, American College of Medical Genetics; P, Pathogenic; LP, Likely Pathogenic; PGD, Preimplantation Genetic Diagnosis; PND, Prenatal Diagnosis. Figure created using BioRender.com.

**Extended Data Fig. 6:**
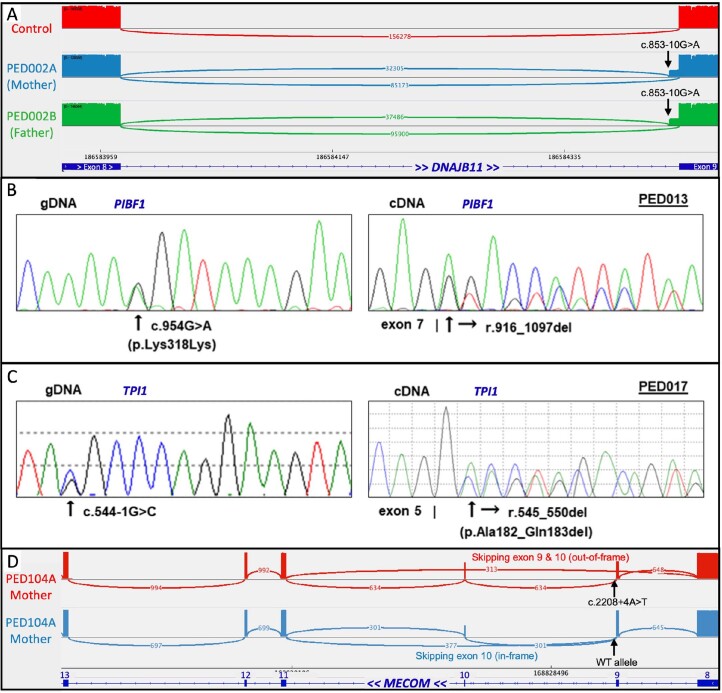
Evaluation of potential splice effects by RT-PCR and Sanger or Nanopore sequencing of PED002, PED013, PED017 and PED104. RNA analysis for interpretation of variant effect in PED002, PED013, PED017 and PED104. **(a)** RT-PCR and nanopore sequencing results for PED002A (mother; blue) and PED002B (father; green) versus a control blood sample (red). The Sashimi plot shows retention of 8 intronic bases (black arrow) of the 8th intron of DNAJB11 as a result from the intronic c.853-10 G > A variant in both parents. **(b)** RT-PCR and Sanger sequencing of paternal cDNA shows the synonymous PIBF1 c.954 G > A p.(Lys318 = ) variant identified in PED013 causes skipping of exon 8, predicted to result in a downstream frameshift and premature termination. The right (cDNA) figure shows the initiation of a heteroduplex after exon 7, with the mutant allele continuing to exon 9. **(c)** RT-PCR and Sanger sequencing of fetal cDNA shows the TPI1 c.544-1 G > C variant identified in PED017 alters the canonical splice acceptor site of exon 6, resulting in an in-frame deletion of 2 amino acids. The right (cDNA) figure shows the start of a heteroduplex after exon 5, with the mutant sequence displaying a 6 bp deletion from the start of exon 6. **(d)** RT-PCR and nanopore sequencing on a maternal blood sample of PED104 A shows skipping of the (out-of-frame) exon 9 in MECOM as a result from the intronic c.2208 + 4A > T variant (red). The long reads included a synonymous SNP (c.2667 G > A) in exon 14, which allowed separation and comparison of the mutant (red) versus wildtype (blue) alleles.

**Extended Data Fig. 7:**
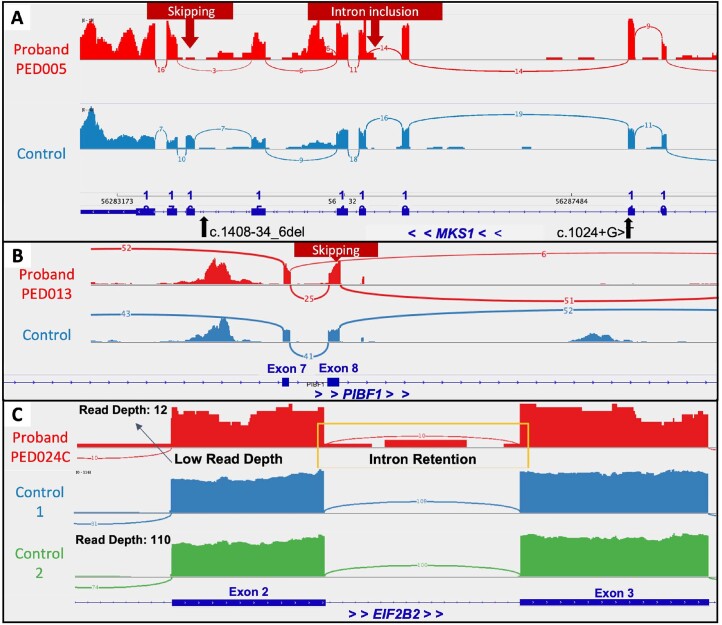
Interpretation of Poly(A) RNA sequencing data for PED005, PED013 and PED024. RNA sequencing analysis for interpretation of variant effect in PED005, PED013 and PED024 **(a)** RNA-seq confirmed that the paternal MKS1 c.1408-34_1408-6del variant results in skipping of exon 16 in the proband (red) versus control (blue) sample. The novel maternal c.1024 + 1 G > T variant revealed altered splicing downstream of exon 11, resulting in partial inclusion of intron 12. **(b)** RNA-seq for PED013, showing skipping of exon 8 in the proband sample (red) versus control (blue) as a result from the synonymous PIBF1 c.954 G > A p.(Lys318=) variant. **(c)** RNA-seq results for PED024, showing retention of intron 2 (yellow box) in the proband sample (red) verus control samples (blue and green), and much lower expression values which are likely due to nonsense mediated decay as a result from the intronic EIF2B2 c.284 + 5 G > T variant.

From the mendeliome analysis of trio/quad exome or genomes, based on OMIM disease genes with clinical presentations completely or partially concordant with the known prenatal and/or postnatal phenotypic spectrum, an American College of Medical Genetics (ACMG)-classified pathogenic (P) and likely pathogenic (LP) variant was identified in 42 of 200 families (21.0%) (Fig. 1, Table 1 and Supplementary Table 2)^14,15^. For an additional 10 of 200 families with ACMG-classified variants of uncertain significance (VUS), a definitive genetic diagnosis was achieved via additional genetic or experimental evidence from identification of unrelated kindreds, RNA analyses, gene-specific in vitro studies and mouse models (Table 1, Extended Data Figs. 5–7 and Supplementary Tables 2–4 (refs. ^16–19^). In 10 of 52 solved cases (19.2%), the proband presented a phenotype expansion of earlier (in utero) or severe presentations previously undescribed/unrecognized within the clinical spectrum of well-studied genomic disorders (Supplementary Table 2). Phenotype expansions were mostly observed in patients with autosomal recessive (AR; *n* = 6) or X-linked recessive (XLR; *n* = 2) variants (Supplementary Table 1).

**Table 1:** Diagnoses from genomic autopsy. All ACMG-classified likely pathogenic (LP) and pathogenic (P) variants identified in the cohort, including follow-up investigations to confirm causality of eight VUS and two GUS. AR, autosomal recessive; AD, autosomal dominant; XLR, X-linked recessive; hom, homozygous; ch, compound heterozygous; dn, de novo; mat, maternal; pat, paternal; (2), two variants with the same inheritance model

PED ID	Proband, major organ system	Inheritance	Gene symbol, amino acid or cDNA change(s) (ACMG classification) and ^follow-up to reclassify VUS^
001	Skeletal	AR-hom	*FGFR2* p.R255Q (LP) ^in vitro^
002	Urogenital	AR-hom	*DNAJB11* c.853-10 G > A (LP) ^RNA^
005	Neurological, urogenital	AR-ch	*MKS1* c.1024 + 1 G > T (P)/c.1408-34_6del29 (P) ^RNA^
007	Lymphatic	AR-ch	*MDFIC* p.M131fs* (P)/p.F244L (LP) ^RNA, in vitro, in vivo, kindreds^
008	Metabolic, endocrine	AD-pat	*ABCC8* p.L1543P (P)
012	Skeletal	AR-ch	B3GAT3 p.R169W (P)/p.R225*(P) ^in vitro^
013	Neurological	AR-ch	*PIBF1* c.954 G > A (LP)/p.K353fs* (P) ^RNA^
014	Lymphatic, skeletal	AD-dn	*RIT1* p.A57G (P)
017	Hematopoietic, immune	AR-ch	*TPI1* p.E105D (P)/c.544-1 G > C (P) ^RNA^
018	Neurological	AD-dn	*ARID1B* p.W1499* (P)
023	Neurological	AR-ch	*NDE1* p.T243Pfs* (P)/p.L245Pfs* (P)
024	Neurological	AR-ch	*EIF2B2* c.284 + 5 G > T (P)/p.R172* (P) ^RNA^
031	(Neuro-)Muscular	AR-ch	*NEB* p.M4377Kfs* (P)/p.H6907Ifs* (P)
033	(Neuro-)Muscular	AD-dn	*NALCN* p.L1324F (LP)
042	Skeletal	AR-hom	*CANT1* p.E215K (LP) ^in vitro^
043	Urogenital	AD-pat^10–20%^	*PBX1* p.R107W (LP)
044	Cardiovascular	AD-dn	*GNB2* p.K89E (P) ^kindreds^
046	Cardiovascular	AD-dn (2)	*KMT2D* p.L2331* (P); *SOS2* p.T295A (LP)
053	Urogenital	AD-dn	*HNF1B* c.344 + 2_5delTAGG (P)
054	Global	AD-dn	*ARID1A* p.A1136S (LP)
056	No abnormality detected	XLR	*ARSL* p.R403* (LP)
057	Skeletal	AD-dn	*NIPBL* p.Y2430C (P)
063	Metabolic, endocrine	AD-dn	*SAMD9* p.R824Q (P)
069	Skeletal	XLR-dn	*HUWE1* p.L2176R (LP)
074	Global	AD-dn	*SMARCB1* p.R377H (P)
084	Neurological	AD-pat^2–3%^	*TUBA1A* p.R64W (P)
085	Global	AD-dn	*RAF1* p.L613V (P)
086	Urogenital	AD-dn	*GREB1L* p.T1872Nfs* (P)
091	Respiratory	AD-dn	*MAP2K2* p.E207A (LP)
093	Hematopoietic, immune	AD-dn	*PTPN11* p.T73I (P)
098	Global	AR-ch	*POLG* p.R309H (P)/p.A467T (P)
103	Neurological	AD-dn	*DDX3X* p.P568L (P)
104	Hematopoietic, immune	AD-mat (2)	*MECOM* c.2208 + 4 A > T (LP) ^RNA^; *RYR2* p.V4176M (LP)
116	Skeletal, cardiovascular	AR-ch	*ADAMTSL2* p.R113H (P)/p.G354Afs* (P)
119	Skeletal, lymphatic	AD-dn	*LZTR1* p.G301V (LP)
121	Pulmonary, cardiovascular	AD-mat	*FOXF1* p.G302Pfs* (P)
138	Urogenital	AR-hom	*PKHD1* p.R564* (P)
156	(Neuro-)Muscular	AD-dn	*ACTA1* p.E272K (LP)
157	Neurological	AD-dn	*USP9X* p.F208Cfs* (P)
158	Neurological	AD-dn	*ACTA1* p.G199D (LP)
165	Urogenital	AD-dn	*USP9X* p.Q2246* (P)
169	Neurological	AR-hom	*TRAPPC12* p.Q149* (P)
176	Urogenital	AD-dn	*GATA3* p.C285Y (LP)
178	Urogenital	AD-dn	*ARCN1* p.R170* (P)
187	Neurological	AD-dn	*TUBA1A* p.R79C[P]
189	Skeletal	AD-dn	*NIPBL* p.D1991N (LP)
199	Cardiovascular	AD-dn	*PRKACB* p.H135L (LP)
200	Neurological	XLR	*ARSL* p.E407* (LP)
207	Global	AD-dn	*KMT2D* p.Q2014* (P)
226	Cardiovascular	AD-dn	*FGFR2* p.P253R (P)
231	Neurological	AR-ch	*OSGEP* p.R186* (LP)/p.R247Q (P)
233	Other	AD-mat	*COL2A1* p.C1289Pfs* (LP)

The majority (57.7%, 30 of 52) of variants leading to definitive diagnoses occurred de novo in the proband (Fig. 1), including one dual de novo diagnosis of Kabuki and Noonan syndromes in PED046, comparable to diagnostic yields in other nonconsanguineous study cohorts^20^. A further one-third (30.8%, 16 of 52) were AR (five homozygous and 11 compound heterozygous) while the remaining were autosomal dominant (AD) with reduced penetrance (7.7%, 4 of 52) or XLR (3.85%, 2 of 52) (Fig. 1, green). While the percentage of selected candidates (VUS/genes of uncertain significance (GUS)) was 27% in all three subgroups, the yield of LP/P variants was highest (45%) in the spontaneous neonatal death group compared with terminated pregnancies (24%) and spontaneous fetal death in utero (19.5%) (Extended Data Figs. 1–4).

### Identification of candidate variants from ‘research’ analysis

In addition to the 52 families with a molecular diagnosis from mendeliome analyses or functional validation, a further 53 families were triaged into the research setting for the following: (1) novel variants (17 of 53) or phenotypes (ten of 53) previously undescribed in well-established OMIM disease genes, and (2) predicted deleterious variants (26 of 53) in GUS with none to limited gene–disease relationships. The 53 candidates included 15 AD de novo variants, 26 AR variants (eight homozygous and 18 compound heterozygous), seven XLR variants, three AD variants with reduced penetrance and a single case with suspicious digenic variants (Fig. 1, yellow). For the remaining 47.5% (95 of 200) of families, no diagnostic or research candidate was identified from the exomes or genomes with supporting evidence available at the time of analysis (Fig. 1, white).

Next we prioritized candidate variants in 33 families, in genes with none to limited gene–disease relationships to the phenotype described in the proband, for further investigation, with the intent of reclassification and issuing a research report to supplement the diagnostic (mendeliome) report. These included variants in potentially novel disease genes (*n* = 18) exhibiting novel phenotypes (*n* = 15) yet to be described in the literature. For variants prioritized from research analysis, 66 in 50 genes were shared on gene-matching platforms^21,22^ yielding 11 matches with relevant genotype–phenotype overlap (Supplementary Table 4). Two of these matched variants (compound heterozygous variants in *MDFIC* (PED007) and a de novo *GNB2* variant in PED044) have recently been published and are now considered solved^16,17^. Collaborative patient cohort collection and experimental follow-up studies are currently ongoing for candidate variants in an additional ten genes.

### Extended cohort analyses

**Fig. 2:**
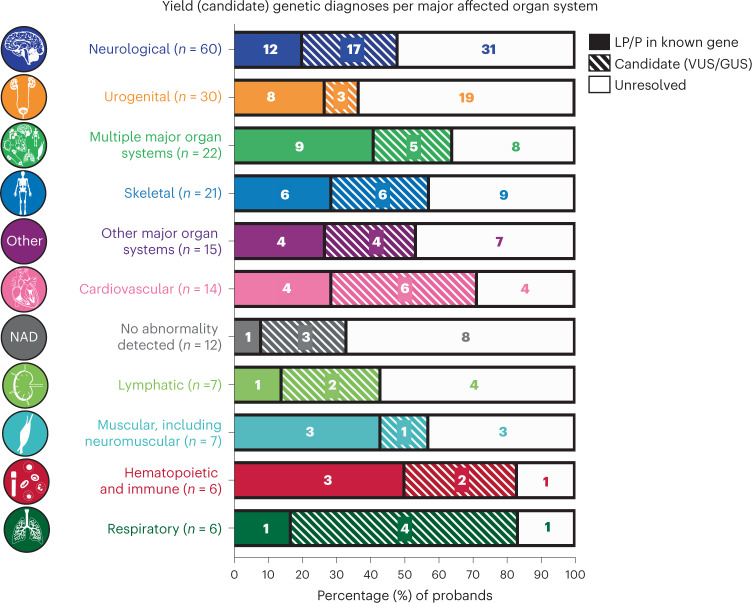
Diagnoses per major organ system affected. Distribution of probands and percentage (candidate) diagnoses per major affected organ system based on ACMG classification. LP, likely pathogenic; P, pathogenic; VUS, variant of uncertain significance; GUS, gene of uncertain significance (that is, novel gene); filled color, LP/P variant; hashed color, candidate variant; no color, no variant. Icons adapted from BioRender.com.

Following mendeliome analysis alone, 79% (158 of 200) of families did not receive a definitive diagnosis, of which 47.5% (95 of 200) remained without a selected candidate for follow-up. The lowest diagnostic yield was observed for cases of perinatal death without any congenital abnormalities, of which 91.7% (11 of 12) remained without a diagnosis (eight of 12, 66% without a candidate) (Fig. 2 and Supplementary Table 2). Notably, despite previous reports suggesting that variants in ‘sudden death’ genes contribute to unexplained stillbirths, we identified only one inherited candidate variant (VUS) in KCNJ2 (PED049) in our cohort, with no definitive diagnoses (LP/P variants) detected in these genes^23,24^.

When looking at the overall contribution of genetic variants identified in our cohort, only seven genes (*FGFR2*, *KMT2D*, *ARSL*, *TUB1A1*, *NIPBL*, *USP9X* and *ACTA1*) were found to be recurrently mutated, with variants in the remaining 96 being observed only once in our cohort (Supplementary Table 2). Families clinically and/or molecularly identified as being related/consanguineous were more likely to yield a (candidate) genetic diagnosis compared with families that were unrelated: 75.0% compared with 50.5% (not statistically significant; Extended Data Fig. 9a). Additionally, disease-causing variants were identified more in female probands (33%, 32 of 97) compared with male probands (19.4%, 20 of 103) (Fig. 1 and Supplementary Tables 1 and 2). Although identification of (likely) pathogenic variants in families with recurrent perinatal death was higher (31.6%) compared with isolated cases (24.5%), the number of LP/P findings from families analyzed as quads was low (14.3%). Of 21 quads, in 14 (66.7%) families both siblings were male compared with five families with siblings of both sex (23.8%) and two (9.5%) with both females (Extended Data Fig. 9b).

### Management of incidental findings

While we did not routinely analyze parental genomic data for potential predisposition to late-onset disorders, there have been limited cases where the proband has inherited a (likely) causative variant from either parent, which may lead to late-onset disease in presently healthy heterozygous carriers (such as the variants affecting DNAJB11 in PED002 and COL2A1 in PED233). These (likely) causative variants were reported back to the referring clinical team recommending both genetic counseling and segregation in extended family members.

### Interpretation of variant effect from RNA analysis

PCR with reverse transcription (RT–PCR) in combination with Sanger or Nanopore sequencing, or Poly(A) RNA-seq was performed as an adjunct test in seven selected cases to support the interpretation of uncharacterized splice-site variants, a synonymous variant and a frameshift variant in known and novel disease genes. This analysis facilitated variant reclassification as LP/P by demonstrating intron retention (PED002, PED005, PED024) or exon skipping (PED005, PED013, PED104) resulting in a frameshift and subsequent premature termination codon, or showing in-frame deletion of two amino acids (PED017) (Extended Data Figs. 6 and 7). RNA analysis for PED007 also demonstrated that the frameshift allele was not subject to nonsense-mediated decay^17^.

### De novo follow-up by phasing and droplet digital PCR

**Extended Data Fig. 8:**
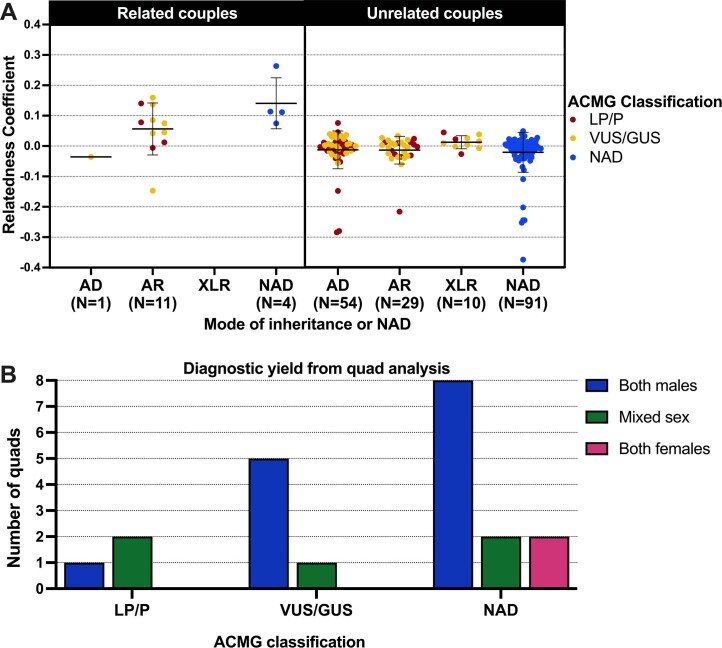
Relatedness and quad analysis diagrams. **(a)** Boxplot shows the relatedness coefficients (y-axis) of all families (dots) and their diagnostic findings based on mode of inheritance (x-axis). The relatedness coefficients were calculated using Peddy^48^, and required a minimum of 1000 shared heterozygous alternate calls per sample. This boxplot indicates that families that are related are more likely to yield a (candidate) diagnosis (12/16, 75%), compared to unrelated families (93/184, 50.5%), not statistically significant (P value = 0.0710, Two-sided Fisher’s exact test). **(b)** Diagnostic yield from families with two affected individuals. This bar plot shows the sex of the two affected individuals per family and the exome findings categorised by their ACMG classifications. There is a slightly higher proportion of families, where both affected individuals are males, with no diagnostic finding (8/14, 57.1%) versus mixed sex siblings (2/5, 40%). Abbreviations: ACMG, American College of Medical Genetics; AD, Autosomal dominant; AR, Autosomal recessive; XLR, X-linked recessive; NAD, no abnormalities detected; LP/P, likely pathogenic or pathogenic; VUS, variant of uncertain significance; GUS, Gene of uncertain significance.

**Extended Data Fig. 9:**
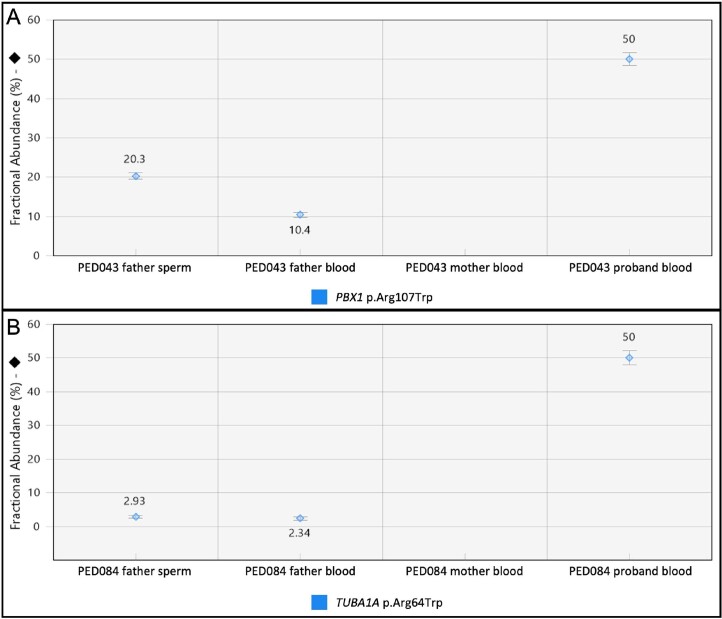
Interpretation of droplet digital PCR results for parents with mosaicism >1%. **(a)** ddPCR shows the PBX1 p.Arg107Trp variant in PED043 is present at different allelic ratios in paternal sperm (20.1%) compared to paternal blood (10.4%). The mother does not carry the variant and s and the proband is heterozygous. **(b)** ddPCR of the TUBA1A p.Arg64Trp variant in PED084 is present at an allelic ratio of 2.9% in paternal sperm and at 2.3% in paternal blood. The maternal sample is negative for the mutation, and the proband is heterozygous. Figures are generated by the original Quantasoft^TM^ software for ddPCR analysis (BioRad). The concentration (copies/µl) of the WT and the Mutant allele are used to calculate the fractional abundance (mean with 95% CI).

Systematic follow-up of identified de novo variants was performed to more accurately define recurrence risk. Phasing based on ES data or Nanopore sequencing showed that 76.5% (26 of 34) of autosomal de novo variants occurred on the paternal allele while only one out of four (25%) X-chromosomal de novo variants was traced to the paternal allele. Custom droplet digital PCR (ddPCR) showed (low-level) parental postzygotic mosaicism in blood for 4 of 43 (9.3%) Genome Analysis Toolkit (GATK)-called de novo variants. For two of these (PED043 and PED084), paternal sperm DNA showed increased variant allele frequencies of the disease-causing de novo variants, thereby redefining recurrence risk to 20.1 and 3.0%, respectively^25^ (Supplementary Tables 2 and 6 and Extended Data Fig. 8).

### Reproductive outcomes following diagnosis

**Fig. 3:**
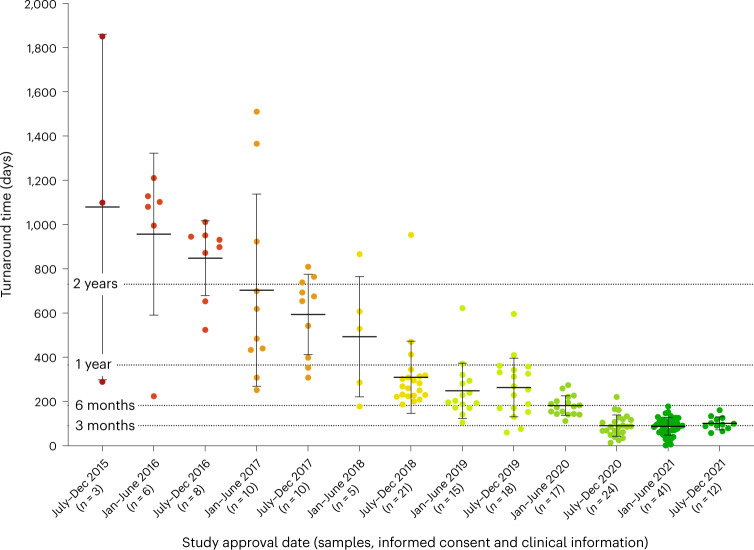
Turnaround between consent and reporting. Reduction in turnaround times as our research study progressed towards a diagnostically accredited test for the genomic investigation of perinatal death. For each specified time range, the number of probands and mean with s.d. of turnaround times are provided. Each dot represents one proband. *n*, number of probands.

We report that definitive and candidate diagnoses provided by genomic autopsy were clinically utilized in the reproductive planning of ten families (five for PGD resulting in transfer of an unaffected embryo and five for PND; Table 2). Recurrent pregnancy loss, termination or perinatal death was experienced by 22.5% (45 of 200) of families. Combining this recurrence rate with the information that at least 24 couples conceived a subsequent pregnancy before receiving a result from genomic analysis (Supplementary Table 2) provided impetus to reduce turnaround times for improvement in clinical utility. We therefore adjusted our workflows and protocols to provide clinically accredited reports within three months (Fig. 3). From five separate pregnancies, for four couples utilizing PGD five healthy babies have already been delivered. Notably, after four consecutive affected pregnancies the couple in family PED005 were able to use PGD to facilitate the birth of two unaffected children (Table 2). A fifth couple did not become pregnant following transfer of the single unaffected embryo (Table 2). The remaining five families chose to use the information for PND, via chorionic villus sampling at 10–13 weeks’ gestation, to enable relatively early identification of potential recurrence. From six separate pregnancies four healthy babies have been delivered, although one child does carry the familial mutation for a disorder known to have variable penetrance. In the remaining two pregnancies the fetuses were found to carry the causative mutations and, in both cases, the parents elected to continue the pregnancy with informed management (Table 2). For another 28 families with autosomal or X-linked de novo mutations (LP/P), the recurrence risk of 1% provided relief and reproductive confidence for future pregnancies.

**Table 2:** The genomic outcomes of our study have informed management for 12 future pregnancies in 10 families, of which 5 elected preimplantation genetic diagnosis (PGD) and 5 had prenatal diagnosis (PND). AR, autosomal recessive; AD, autosomal dominant; XLR, X-linked recessive; hom, homozygous; ch, compound heterozygous

PED ID	Genomic autopsy outcome	Inheritance (gene)	Reproductive history before genomic autopsy	Assisted reproductive choice	Outcome
001	Solved	AR-hom (*FGFR2*)	One affected pregnancy	PND (×1)	One affected pregnancy (liveborn)
002	Solved	AR- hom (*DNAJB11*)	Two affected pregnancies	PGD (×1)	One unaffected child
005	Solved	AR-ch (*MKS1*)	Four affected pregnancies	PGD (×2)	Two unaffected children
013	Solved	AR-ch (*PIBF1*)	One affected pregnancy	PGD (×1)	Single unaffected embryo failed to implant
017	Solved	AR-ch (*TPI1*)	One affected pregnancy	PGD (×1)	One unaffected child
040	Candidate	AR-ch (*LAMC3*)	One affected pregnancy	PND (×2)	Two unaffected children
043	Solved	AD, paternal mosaic (*PBX1*)	One affected pregnancy	PND (×1)	One affected pregnancy (stillborn)
051	Candidate	AD, variable penetrance (*ZFPM2*)	Two affected pregnancies; maternal grandmother and great-uncle also affected	PND (×1)	One unaffected child (but has familial variant)
056	Solved	XLR, maternal (*ARSL*)	Two affected pregnancies	PGD (×1)	One unaffected child (girl)
098	Solved	AR-ch (*POLG*)	Two affected pregnancies	PND (×1)	One unaffected child

## Discussion

The clinical efficacy of an ES or GS approach for the molecular diagnosis of postnatal developmental disorders is well documented, with diagnostic yields ranging from 10 to 70% dependent on the primary presentation^20,26^. As a result, rapid genomic testing is implemented for neonates and pediatric patients to guide therapeutic intervention in an acute care setting^27^. More recent studies have also focused on fetuses with congenital abnormalities identified on ultrasound, and have implied that quick turnaround times may aid couples when decisions on elective terminations of pregnancy are to be made^28–30^. However, to our knowledge, no studies have reported the clinical importance of reducing turnaround times for ES and GS in the absence of alive patients or fetuses, and genomic analyses focusing on fetal and neonatal cases at the lethal end of the spectrum have not yet been implemented in the clinical setting. We show that selection for severe (lethal) cases, collection of detailed phenotypic information at autopsy and the addition of follow-up investigations for VUS and GUS result in an increased diagnostic yield (26%) compared with studies focusing on structural abnormalities on ultrasound (8.5–10.0%)^28,29^.

A small number of research projects have sought to elucidate the genetic causes of pregnancy loss, terminations of pregnancy and perinatal death by applying genomics, returning diagnostic yields ranging from 14 to 57% (refs. ^31–37^), suggesting that many of these cases will have an identifiable monogenic basis. While differences in study design and inclusion criteria probably account for some of the variability in diagnostic rate, this discrepancy also reflects the challenges currently faced in the genomic analysis of disorders presenting prenatally. Due to the skewing of existing reference datasets to postnatal—and particularly adult—profiles of gene function and expression, interpretation and classification of genetic variants in a prenatal versus postnatal setting is often more challenging due to limitations of in utero phenotyping^14,38,39^ and availability of appropriate databases and guidelines for fetal classifications^14,40^. Probably influenced by these factors, the genetic diagnostic yield (LP/P variants) was highest in our spontaneous neonatal death subgroup (45%). Despite previous molecular investigation of suspected disorders based on phenotype, 62% (53 of 105) of (candidate) diagnoses were made in known OMIM disease genes, with retrospective phenotype review revealing substantial overlap between the proband and reported phenotype. A further 12.4% (13 of 105) of (candidate) diagnoses represented novel phenotype associations with known disease genes, and the remaining 25.7% (27 of 105) were made in potentially novel disease genes (further details provided in Supplementary Tables 1 and 2).

Within our cohort, the phenotypic distribution ranged from severe congenital abnormalities to unexplained fetal loss. While the cases in our study were consecutively referred from different hospitals, there are some inherent limitations in seeing this group as representative of a population of pregnancy loss and perinatal death. Compared with the overall distribution of clinical subtypes of pregnancy loss, pregnancy terminations and perinatal death^11^, this cohort is skewed towards terminated pregnancies of fetuses with congenital abnormalities, and the reported major organ systems (Fig. 2 and Extended Data Figs. 1 and 2) are enriched for essential organs detected on early fetal ultrasound (that is brain, urogenital, skeletal and cardiovascular). After exclusion of cases diagnosed by karyotyping, single-nucleotide polymorphism (SNP) arrays or gene panels that are part of standard-of-care in Australia, the genomic analyses also indicated a large heterogeneity in the genetic defects underlying perinatal death, with only seven recurrently mutated genes reported in our cohort. While dual diagnoses are expected to contribute to (phenotype expansions of) developmental disorders in 5% of cases^41^, reportable candidate variants in two genes were identified in only three families without phenotype expansions. In addition, candidates with possible digenic inheritance were detected in one family with a specific clinical presentation.

The majority of definitive diagnoses occurred de novo in the proband, while the majority of candidate variants were autosomal or XLR (Fig. 1). This variation in distribution in causative and candidate variants can partially be explained by the additional weight of the criteria for de novo variants (PS2/PM6) in the ACMG guidelines^15^. Parents are currently counseled that a recurrence risk for de novo variants is ~1%. However, systematic follow-up of de novo variants by ddPCR revealed that two of these were paternal mosaic at allele balance >1% (PED043 and PED084) and two others were detectable at low level (<1%), leaving 39 ‘true’ de novo (candidate) variants that probably arose during gametogenesis.

Surprisingly, we identified more LP/P variants in female probands compared with male (Fig. 1 and Supplementary Tables 1 and 2). This may be explained partially by the higher proportion of more mature females recruited (34 females recruited in the last trimester of pregnancy compared with 21 males; Extended Data Fig. 1). Fetuses of greater maturity are more likely to have a phenotype that is recognizable and more comparable to the phenotypic features in a neonate, making interpretation of variants easier. There is also a higher number of de novo mutations in known disease genes in female (20) compared with male (10) probands in the cohort. While the diagnostic rate (LP/P variants) in families with recurrent perinatal death was higher compared with isolated cases, the number of LP/P findings from families analyzed as quads was unexpectedly low (14.3%), indicating that probable autosomal or XLR genetic variants were not detected (Supplementary Table 1).

The interpretation, classification and reporting of VUS and GUS remains challenging in the clinical setting. Our integrated research follow-up investigations confirmed pathogenicity of 15.9% (10 of 63) of all selected VUS/GUS, aiding in reclassification of these variants (Table 1 and Supplementary Table 3), which is one of the strengths of our approach. Despite the diagnostic impact, analysis for GUS and experimental follow-up of VUS/GUS variants is laborious, preventing uptake in routine clinical genomic analysis.

Considering the phenotypic heterogeneity of the study cohort and the high proportion (23%, 24 of 105) of candidate variants in (potentially) novel disease genes^16,17^, follow-up research will be instrumental in providing additional diagnoses in cases that remain unresolved following clinical genomic analyses. In collaboration with other fetal cohorts, this research will support the characterization of all fetal lethal genomic disorders resulting from deleterious variants in genes intolerant to variation (that is, the intolerome)^37^. Candidate variants in potentially novel disease genes from this study were prioritized by haploinsufficient genes, based on the loss-of-function observed/expected upper-bound fraction (LOUEF) scores that measure gene constraint, and phenotypic overlap (for example, early lethality) with mouse knockout models and/or additional kindreds from gene-matching approaches (Supplementary Table 4). For the remaining families with no diagnostic or research candidates, periodic reanalysis in the research phase via alternative sequencing technologies, analytical approaches or tissue source can ensure that diagnoses are not missed due to technological limitations, or to lack of literature evidence for variant interpretation at the time of mendeliome analyses. Additionally, the placental genome remains an underexplored area in the understanding of spontaneous pregnancy loss and is an ongoing focus of our research^42^.

The phenotype expansions observed in our cohort include severe prenatal presentations of postnatal disorders and additional clinical manifestations within the same organ system and other affected organ systems. In cases with phenotype expansions, ES or GS did not identify (candidate) variants that would explain the additional phenotypic features. Nevertheless, we cannot exclude the possibility that (modifying) variants were missed either by the sequencing method or in analysis. Interestingly, phenotype expansions were mostly observed in patients with autosomal or XLR variants and were generally more severe compared with those reported in the literature (Supplementary Table 2; PED012, PED042, PED043)^18,25^, which may be explained by the resulting variant impact—that is, complete loss-of-function versus reported missense variants that may be partial loss-of-function or hypomorphic variants. However, other cases (PED056 and PED200) did not show phenotypic features consistent with X-linked chondrodysplasia punctata, and (standard-of-care) targeted genetic testing would not have included the ARSL gene.

Our results strongly support a clinical role for genomic testing in elucidating the cause of pregnancy loss and perinatal death, particularly when congenital abnormalities are present. In 21% of cases, genomic autopsy provided a clear diagnosis where standard autopsy could not. Despite this, standard autopsy remains a valued assessment tool in perinatal death due to its ability to identify nongenetic cause(s) of death (for example, congenital abnormalities of diabetic embryopathy or evidence of cytomegalovirus infection). In addition, the anatomical and histological phenotypic information obtained at autopsy is more detailed (HPO terms given in Supplementary Table 2) compared with that identified on fetal ultrasound^28,29^, further improving our interpretation of candidate variants and allowing phenotypic review following identification of variants. Therefore a genomic autopsy is best implemented alongside current standard-of-care measures to help improve diagnostic rates for perinatal death.

Although tracing the ultimate reproductive outcomes for women/couples was not the primary focus of this study, following our genomic findings at least ten families are known to have used the information for reproductive planning, with five electing for PGD and five for PND, facilitating nine healthy pregnancies, one embryo that failed to implant (PGD) and two early detections of recurrence (PND) (Table 2). A particularly impressive example of the utility of molecular diagnosis is family PED005, who were able to use PGD to facilitate the birth of two unaffected children. Two families in this study chose to use PND (PED040, two pregnancies and PED051, one pregnancy) based solely on candidate variants (VUS)^43^, resulting in the birth of three unaffected children, although one of these (PED051) carries the familial variant that is known to present with variable penetrance (Table 1). However, these motivated families were extensively counseled because the impact of candidate variants (VUS) remains uncertain and the clinical use (PND) may provide a false sense of reassurance.

The diagnostic yield in our cohort provides evidence that genomic autopsy in the investigation of pregnancy loss, pregnancy terminations and perinatal death is advantageous over currently performed diagnostic tests. We conclude that the addition of a genomic approach to the first line of investigation in the clinical standard-of-care for fetal and neonatal loss will provide more families with answers, leading to reproductive confidence (for de novo variants) or enabling options to prevent recurrence (for inherited variants).

However, in addition to diagnostic utility, an integrated clinical–diagnostic research setting remains beneficial because pregnancy loss and perinatal death represent an understudied heterogeneous cohort and genetic causes of early lethality remain to be discovered. The research pathway to discovery includes large-scale data aggregation of fetal cohorts^44^, systematic functional assays to prove causality of rare variants, testing for genetic mosaicism in the most affected tissues and application of the latest genomic technologies to detect a broader range of genetic variation.

## Methods

### Ethics declaration and consent to participate

This study was performed as part of the NHMRC- and GHFM-MRFF-funded Genomic Autopsy Study and was approved by the Human Ethics Committee of Central Adelaide Local Health Network (no. R20160813), the Women’s and Children’s Health Network, South Australia, Australia (no. HREC/15/WCHN/35) and the Melbourne Health Human Research Ethics Committee as part of the Australian Genomics Health Alliance protocol (no. HREC/16/MH/251). Informed consent for genomic analysis and participation in study protocols was obtained from all participants, including biological and gestational parents, and all research was conducted in accordance with the Declaration of Helsinki. Parents consented to the use of their samples, and the samples of their child retained at autopsy, for the purpose of trying to identify the cause of their pregnancy loss or perinatal death. Consent was also obtained to allow access to relevant medical information and subsequent storage of anonymized samples, genomic data and/or medical information in relevant databases and publications. Study data were collected and managed using REDCap electronic data-capture tools, which is a secure, web-based software platform designed to support data capture for research studies^45,46^. Participants did not receive compensation for their involvement, but were not charged for any testing undertaken as part of the research study. Use of genomic findings in future reproductive planning, and subsequent genomic testing on embryos, was performed as part of follow-up clinical care, independent of the research study, with outcomes provided back to the research team where the participants remained under the care of the referring clinical teams.

### Study design

The definition of perinatal death varies globally^47^. For this study we included cases of stillbirth and neonatal death occurring between 20 weeks of gestation and 28 days postpartum, as well as euploid miscarriages occurring between 13 and 20 weeks, to reflect all gestations for which a standard-of-care autopsy can be performed^9^. Consistent with Australian definitions, terminations of pregnancy for congenital abnormality were included alongside spontaneous deaths due to congenital anomaly or where death was unexplained. Routine autopsy, including collection of obstetric and family history and anatomical pathology of the fetus and placenta, amongst other investigations, was performed for all cases allowing detailed phenotypic information to be obtained. Microarray was performed for all cases, with single-gene or panel testing performed where indicated by a specific phenotype. For inclusion in the study, previous standard-of-care testing must not have yielded a genetic diagnosis that could explain the disease phenotype. The first 200 consecutively referred families between 2011 and 2021 (179 trios and 21 quads; Supplementary Tables 1 and 2) are included in this study. Sex of the proband was not a determinant for case selection, but was considered to enable appropriate genomic variant analysis (for example, contribution of X-linked variants). Self-reported (parents) or clinically determined (probands and siblings) sex of all study participants was confirmed genomically using Peddy^48^ or Somalier^49^, and established sex is presented throughout.

### Patient population included in the study

While these cases were sequentially referred, they do not represent the complete spectrum of pregnancy loss and perinatal death because only patients within the public healthcare system were recruited and only cases with consent were approved into the study. To evaluate the percentage of cases compared with the fetal and perinatal loss population, we reviewed 1 year of clinical data. In that year (2017), 234 local cases of fetal loss and perinatal death were discussed during weekly multidisciplinary team (MDT) meetings involving several health professionals and specialists at the Women’s and Children’s hospital in Adelaide (with 97 families the main referral site for our study, and the only one where we have insight into these data). Of these 234 cases, 80 were assessed as being potentially eligible for our study, 31 were referred and 18 were recruited. From this review, we conclude that clinically biased ascertainment is unlikely because of a thorough assessment of cases at an MDT meeting, more families are recommended for referral than are actually referred (from 2017 about 40% who could potentially have been referred were referred) and, once referred, over half were recruited (60% in 2017). There is no clinical selection bias except potentially obstetrician-gynecologists not referring, which would be random. Initially our study focused on the analysis of a select cohort of families who had experienced pregnancy loss, terminations or perinatal death. As our study progressed recruitment became contemporaneous and families were recruited nationwide, resulting in a larger group of referred and consented cases. Further information on proband sex, age (gestation) and reason for referral can be found in Supplementary Tables 1 and 2.

### Nucleic acid isolation

Parental DNA was isolated from whole blood (*n* = 268), saliva (*n* = 116) or an unspecified sample source (*n* = 16). The majority of fetal DNA samples were isolated from unspecified tissue (*n* = 58) followed by lung (*n* = 44), whole blood (*n* = 27), skin (fibroblasts) (*n* = 20) and amniotic fluid (16), with smaller numbers isolated from liver (9), muscle (7), chorionic villus (6) and umbilical cord (6) and single samples from cartilage, cerebellum, cord blood, gonad, kidney, ovary and placenta (Supplementary Table 5). In families with de novo mutations (PED043, PED084, PED095, PED118 and PED128), DNA was also isolated from paternal sperm for use in ddPCR assays. DNA was extracted using the QIAamp DNA Mini Kit (Qiagen, no. 51306) following the manufacturer’s protocol. For selected pedigrees, RNA was isolated from either fetal lung, fetal kidney, cultured fibroblasts or parental whole blood for RNA-seq or RT–PCR analysis. RNA was extracted using the RNeasy Plus Mini Kit (Qiagen, no. 74134) following the manufacturer’s protocol.

### Genomic sequencing

Genome sequencing was performed at either the Kinghorn Centre for Clinical Genomics Sequencing Laboratory (five families), Illumina (two families) or the Australian Genome research facility (one family). DNA was prepared using Illumina HiSeq X Ten chemistry, and libraries sequenced on an Illumina HiSeq X Ten as 150-base pair (bp) paired-end reads. Exome sequencing was performed for 98 families at the Centre for Cancer Biology Australian Cancer Research Foundation (ACRF) Genomics Facility (Adelaide, South Australia, Australia). Exonic sequences were enriched using the Roche SeqCap EZ Exome Library (11 families) and IDT xGen Exome (87 families), and libraries were sequenced as either 100-bp paired-end reads on an Illumina HiSeq 2500 or 150-bp paired-end reads on an Illumina NextSeq 500. For 90 families, ES was performed at the Broad Institute’s Genomics Platform (Cambridge, MA, USA). In brief, exome libraries were created with either a custom Illumina exome capture (38-Mb target; 38 families) or a custom TWIST exome capture (37-Mb target; 52 families) and sequenced as 150-bp paired-end reads on either an Illumina HiSeq 2500 or Illumina NovaSeq 6000, respectively.

### Sequence mapping, variant calling and annotation

Genome sequencing data were processed at the Centre for Cancer Biology ACRF Genomics Facility. ES data was processed at both the Centre for Cancer Biology ACRF Genomics Facility and the Broad Institute’s Data Sciences Platform. Both centers use a pipeline based on GATK best practices (v.3), with mapping to the GRCh37 human reference genome (b37+decoy) performed using BWA mem (v.0.7.12). Sambamba (v.0.6.5) was used for marking PCR duplicates. ES resulted in an average coverage of 96.0-fold, with 94.0% of bases in the targeted region being covered at least 20-fold. GS resulted in an average coverage of 38.4-fold, with 96% of all bases being covered at least 20-fold. SNVs and small indels were jointly called using GATK HaplotypeCaller (v.3.8). Structural variants for GS were called using Manta, and for ES were called using either GATK gCNV (Broad) or an in-house algorithm (ACRF) that normalizes ploidy change across bins optimized for target capture regions. Joint genotyping was then performed using GATK GenotypeGVCF, and variant quality scores were recalibrated using GATK’s VQSR. SNVs and indels were annotated using Variant Effect Predictor (VEP; Broad) or SnpEff (v.4.1; ACRF). CNVs were annotated using AnnotSV, with regions of homozygosity detected by BCFtools.

### Genomic variant analysis

The analysis and interpretation approach utilized in this study is summarized in Extended Data Fig. 5. Analysis of SNVs and indels from ES was performed using both seqr (Broad Institute) and VariantGrid (in-house) analysis platforms, with GS data analyzed solely in VariantGrid. CNVs were analyzed directly from the AnnotSV output. Initial variant filtering selected for rare, protein-altering variants (small variants: gnomAD and in-house frequencies ≤1%, maximum five homozygotes for recessive and ≤0.01%, maximum five heterozygotes for dominant; CNVs: no reciprocal overlap with known benign CNVs ≥70%), consistent with any plausible inheritance model. In families with suspected consanguinity, regions of homozygosity were also annotated. Variants were prioritized for further interpretation based on in silico pathogenicity predictions, sequence conservation scores, protein function and expression and known disease associations (human and animal).

Analysis was performed in two stages: (1) a first-pass mendeliome analysis, looking to identify disease-relevant variants in known disease genes (OMIM-morbid map genes, annotated March 2021 with phenotype-causing mutation), and (2) a second-pass research analysis, looking to identify potentially disease-relevant variants in potential novel disease genes. If no candidate causative variants were identified, filtering and prioritization criteria were relaxed and variants with either lower presumed impact (for example, synonymous and intronic variants) or atypical inheritance mechanisms were considered. All identified variants of interest were manually inspected in the integrative genomics viewer (IGV), with a bed file containing normalized ploidy changes and *z*-score visualized alongside BAM files for CNVs. Candidate causative variants occurring de novo or in regions of low read depth or ambiguous mapping were validated using an orthogonal method, and Sanger sequencing for small variants and microarray or RNA-seq for CNVs. Where DNA samples were available for unaffected or additional affected family members, segregation analysis by Sanger sequencing or microarray was also performed. Variant analysis ended when variant(s) considered causal of the full phenotype were identified.

### RNA analysis for confirmation of splice effects

Quantitative PCR with reverse transcription was performed to aid the interpretation of variant effect (synonymous and splice site) in four selected cases (PED002, PED013, PED017 and PED104). Complementary DNA was generated using the SuperScript III Reverse Transcriptase kit (Invitrogen) following the manufacturer’s instructions, with 150 ng of Random Primers (Promega) and 1 μg of total RNA. The effect of the variant on splicing was assessed by Sanger or Nanopore sequencing of PCR-amplified cDNA (primer sequences are provided in Supplementary Table 7). For Oxford Nanopore Technology (ONT)-sequenced amplicons (PED002 and PED104), libraries were created using a ligation sequencing kit (SQK-LSK109) with barcodes (EXP-NBD104). After sequencing on an ONT Minion flowcell, basecalling was performed using guppy (v.5.0.11) and resultant fastq files were mapped to b37+decoy using minimap2 (v.2.20-r1061). Potential splice effects were visualized and interpreted using IGV.

Poly(A) RNA-seq was performed to aid the interpretation of variant effect in three selected cases (PED005, PED007 and PED024), with candidate causative variants predicted either to affect splicing or result in a prematurely truncated protein product. Analysis was restricted to cases where the gene of interest was expected to be expressed in an available fetal tissue sample. Poly(A) selection of messenger RNA was performed using the NEBNext PolyA mRNA Magnetic Isolation Module. Samples were sequenced as 150-bp paired-end reads on an Illumina Nextseq 500 at the Centre for Cancer Biology ACRF Genomics Facility. Sequence reads were aligned to the b37+decoy human reference genome. Regions surrounding variants of interest were manually evaluated in IGV.

### Identification of additional kindreds

In 33 unrelated families we submitted a total of 39 different variants to Matchmaker exchange through the software Matchbox or GeneMatcher portal^22,50^. These VUS in existing disease genes were submitted for cases requiring phenotype or inheritance expansion, different to the reported evidence and/or for genes with fewer than two reported cases at the time of curation. In addition, GUS with no human disease gene associations, excluding those with susceptibility risks or limited clinical validity (via ClinGen and PanelApp Australia), were submitted based on one or more of the following pieces of evidence: (1) gene-wide or regional constraints, (2) spatial expression in relevant disease tissues or organs and (3) phenotypes from animal models.

### De novo follow-up by phasing and ddPCR

A total of 46 causative and candidate de novo variants in 44 families was followed up by phasing and/or ddPCR (Supplementary Table 6). Phasing of de novo variants was performed depending on the vicinity of informative parental variants: 32.6% (15 of 46) could be phased to either the paternal or maternal allele using existing ES data (150-bp paired-end reads). The 31 de novo variants that could not be phased from ES data were selected for long-range PCR (primer sequences provided in Supplementary Table 7) and ONT sequencing. Unfortunately, insufficient DNA remained to perform long-range PCR on proband samples of four families. For 15 of those families that could be tested, informative SNPs in ES data were selected for primer design and, in a further ten cases, a region of ~5–10 kb around the de novo variant was selected for long-range PCR. Primers were designed using Primer3, and long-range PCR was performed using 2× LongAmp Hot start master mix (New England Biolabs). Samples were pooled in equimolar quantities into three groups (probands, mothers and fathers) before library preparation for ONT. Library preparation was performed using the ligation sequencing kit (SQK-LSK109), and barcodes (from EXP-NBD104) were added per pool (probands NBD-10, mothers NBD-11 and fathers NBD-12). After barcoding, these libraries were pooled and sequenced on a Minion flongle or flowcell (ONT). Mapping of fastq files was performed using minimap2 (v.2.20-r1061). The resulting alignments were visualized alongside ES data within IGV for interpretation.

To assess potential parental mosaicism as the cause of de novo variants, ddPCR was performed as a follow-up test to aid in defining recurrence risk in families with candidate and causative de novo variants. The ddPCR assays were custom designed for each variant and performed using a Bio-Rad QX100 instrument. Genomic locations were provided for each variant, with commercially designed primers and probes supplied by Thermo Fisher Scientific or Bio-Rad. Due to the complexity of either the genomic region or the variant, no ddPCR assays could be designed for three variants in three families (PED046, PED162 and PED187). Assays were performed on DNA from 43 parent–fetal trios, as well as on sperm samples from five of these families, using a Bio-Rad QX100 instrument and analyzed using QuantaSoft (Bio-Rad).

### Variant classification, reporting and diagnostic outcome

All variants of interest were classified according to ACMG guidelines^14,15^, and research or NATA-accredited reports was issued to the referring clinician at the completion of first-pass (mendeliome) analysis. Only variants classified as VUS, LP or P (ACMG class 3–5) and relevant to the proband’s phenotype were reported, with detailed gene- and variant-level curation information included to support interpretation of clinical utility. For VUS in known disease genes, recommendations for further research studies to support causal implication were included (for example, segregation in unaffected family members, in vitro functional studies, animal models; Extended Data Fig. 5). VUS identified in either known disease genes with limited or no phenotype overlap with reported evidence, or in novel disease genes with supporting evidence (gene-wide or regional constraints, spatial expression in relevant disease tissues or organs and phenotypes from animal models), were submitted to Matchmaker exchange to identify additional kindreds. Supplementary research reports were issued to the referring clinician if additional supporting evidence was obtained warranting further investigations (for example, segregation, functional follow-up and so on).

Following completion of analysis, the diagnostic outcome of cases was classified as either ‘unresolved’ (no likely cause identified), ‘candidate’ (VUS variant/s) or ‘solved’ (LP or P variant/s, or VUS variant/s with sufficient evidence to link novel gene to disease). For cases where causative or candidate causative variant/s were identified, the diagnosis was further classified as either ‘known gene’ (proband phenotype predominately consistent with reported phenotype for gene), ‘phenotype expansion’ (proband phenotype notably different to reported phenotype for gene or proband clinical diagnosis not previously linked to gene) or ‘novel gene’ (gene not previously linked to human disease).

### Assessment of phenotype expansions

We have evaluated potential phenotype expansions based on overlapping phenotypes with multiple cases described in OMIM, Clingen gene–disease validity, ClinVar and the literature. Because the defined phenotypic spectrum of genomic disorders is under continuous development, reassessment of genotype–phenotype correlations can be required. For some families, phenotypic features described in both case reports and cases (the supplementary material thereof) from larger cohort studies have resulted in acknowledgement of nonclassical phenotypic features or inheritance modes for genomic disorders. While other features overlap, the timing (1 day after birth) and cause of death (anemia) in PED017 were different from what is known for TPI1 deficiency, commonly resulting in respiratory insufficiency with death at 5–10 years of age.

### Reproductive planning

Consent has been obtained to report outcomes of pregnancy whilst in the standard-of-care under the referring clinical team. Following identification of a diagnosis or candidate diagnosis associated with risk for recurrence, families were counseled regarding reproductive planning, including options for PND and preimplantation genetic testing for monogenic PGD. Families who elected for PGD were referred to appropriate fertility specialists (Monash IVF group).

### Web resources

AnnotSV: https://lbgi.fr/AnnotSV

BCFtools: http://samtools.github.io/bcftools

ClinVar: https://www.ncbi.nlm.nih.gov/clinvar

GATK: https://gatk.broadinstitute.org

gnomAD: https://gnomad.broadinstitute.org

The Human Phenotype Ontology: https://hpo.jax.org/app

IGV: https://software.broadinstitute.org/software/igv

NCBI RefSeq: https://www.ncbi.nlm.nih.gov/refseq

Manta: https://github.com/Illumina/manta

OMIM: https://omim.org

Seqr: https://seqr.broadinstitute.org

SnpEff: http://snpeff.sourceforge.net

VariantGrid: https://variantgrid.com

VEP: https://ensembl.org/info/docs/tools/vep

### Reporting summary

Further information on research design is available in the Nature Portfolio Reporting Summary linked to this article.

## Online content

Any methods, additional references, Nature Portfolio reporting summaries, source data, extended data, supplementary information, acknowledgements, peer review information; details of author contributions and competing interests; and statements of data and code availability are available at 10.1038/s41591-022-02142-1.

## Supplementary information

Reporting Summary

Supplementary Tables 1–7. Supplementary Table 1: Overview of genetic outcomes per subgroup (sex, classification, reason for referral, family structure tested and major affected organ system. Supplementary Table 2: Clinical and parental information and genomic findings (including classification) of all pedigrees in the Genomic Autopsy Study. Supplementary Table 3: Functional follow-up studies to confirm causality of candidate variants. Supplementary Table 4: Candidate variants shared on matchmaking platforms. Supplementary Table 5: Samples used for nucleic acid isolation. Supplementary Table 6: Results from phasing and ddPCR of de novo variants. Supplementary Table 7: Sequences of primers used for long-range PCR and RT–PCR.

## Data Availability

Sequence data have been deposited at the European Genome-phenome Archive, which is hosted by the European Bioinformatics Institute and the Centre for Genomic Regulation under accession no. EGAS00001006295. Controlled access to primary data and/or material generated as part of this study may be requested from the corresponding author (Hamish.Scott@sa.gov.au), and will be shared in a nonidentifiable manner only where participants have consented to the sharing of data and samples for use in ethically approved future research studies. Studies requesting access must produce evidence of appropriate HREC permissions, and detailed description of how the data and samples will be used and stored. Once controlled data access approval has been granted (within 3 months of submission), a material transfer agreement between respective institutions will need to be established. The research team will not accept, or return to participants, any research findings unrelated to the referred condition. All reported variants will be submitted to ClinVar under Molecular Pathology Research Laboratory (organization ID: 507864, pre-2020 research sequenced) and Genetics and Molecular Pathology; SA Pathology (organization ID: 506043, post-2020 clinically sequenced).

## Code availability

The VariantGrid code (www.variantgrid.com) generated during this study (by D.M.L.) is available for research use under business source license 1.1 on GitHub (https://github.com/SACGF/variantgrid). The code for the Seqr platform, used at the Broad Institute, is available at https://github.com/broadinstitute/seqr.
