# Author Correction: Genomic autopsy to identify underlying causes of pregnancy loss and perinatal death

**DOI:** 10.1038/s41591-023-02487-1

**Published:** 2023-07-10

**Authors:** Alicia B. Byrne, Peer Arts, Thuong T. Ha, Karin S. Kassahn, Lynn S. Pais, Anne O’Donnell-Luria, Broad Institute Center for Mendelian Genomics, Alicia B. Byrne, Lynn S. Pais, Anne O’Donnell-Luria, François Aguet, Harindra M. Arachchi, Christina A. Austin-Tse, Larry Babb, Samantha M. Baxter, Harrison Brand, Jaime Chang, Katherine R. Chao, Ryan L. Collins, Beryl Cummings, Kayla Delano, Stephanie P. DiTroia, Eleina England, Emily Evangelista, Selin Everett, Laurent C. Francioli, Jack Fu, Vijay S. Ganesh, Kiran V. Garimella, Laura D. Gauthier, Julia K. Goodrich, Sanna Gudmundsson, Stacey J. Hall, Yongqing Huang, Steve Jahl, Kristen M. Laricchia, Kathryn E. Larkin, Monkol Lek, Gabrielle Lemire, Rachel B. Lipson, Alysia Kern Lovgren, Daniel G. MacArthur, Brian E. Mangilog, Stacy Mano, Jamie L. Marshall, Thomas E. Mullen, Kevin K. Nguyen, Emily O’Heir, Melanie C. O’Leary, Ikeoluwa A. Osei-Owusu, Jorge Perez de Acha Chavez, Emma Pierce-Hoffman, Heidi L. Rehm, Jillian Serrano, Moriel Singer-Berk, Hana Snow, Matthew Solomonson, Rachel G. Son, Abigail Sveden, Michael Talkowski, Grace Tiao, Miriam S. Udler, Zaheer Valivullah, Elise Valkanas, Grace E. VanNoy, Qingbo S. Wang, Nicholas A. Watts, Ben Weisburd, Clara E. Williamson, Michael W. Wilson, Lauren Witzgall, Monica H. Wojcik, Isaac Wong, Jordan C. Wood, Shifa Zhang, Milena Babic, Mahalia S. B. Frank, Jinghua Feng, Paul Wang, David M. Lawrence, Leila Eshraghi, Luis Arriola, John Toubia, Hung Nguyen, Genomic Autopsy Study Research Network, Disna Abeysuriya, Lesley C. Ades, David J. Amor, Susan Arbuckle, Madhura Bakshi, Bligh Berry, Tiffany Boughtwood, Adam Bournazos, Alessandra Bray, Fiona Chan, Yuen Chan, Clara Chung, Jonathan Clark, Jackie Collett, Alison Colley, Felicity Collins, Sandra Cooper, Mark A. Corbett, Jane E. Dahlstrom, Peter Dargaville, Janene Davies, Tenielle Davis, Jarrad Dearman, Jayanthi Dissanayake, Julia Dobbins, Helen Doyle, Andrew Dubowsky, Matt Edwards, Lisa J. Ewans, Mitali Fadia, Andrew Fennell, Keri Finlay, Andrew French, Kathryn Friend, Alison E. Gardner, Jozef Gecz, Nicole Graf, Eric A. Haan, Georgina Hollingsworth, Ari E. Horton, Denise Howting, Matthew F. Hunter, Gareth Jevon, Benjamin Kamien, Debra Kennedy, T. Yee Khong, Michael Krivanek, Thessa Kroes, Emma I. Krzesinski, Edward Kwan, Stephanie Lau, Shannon LeBlanc, Jan Liebelt, Suzanna Lindsey-Temple, Christine K. C. Loo, Julia Low, Amali Mallawaarachchi, Admire Matsika, Tessa Mattiske, Julie McGaughran, Lesley McGregor, Namita Mittal, Ali Moghimi, Lynette Moore, Hatice Mutlu Albayrak, Jessica Ng, Jillian Nicholl, Nicholas Pachter, John Papadimitriou, Renae Parker, Sarah Parsons, Chirag Patel, Rhonda Pawlowski, Luis A. Perez-Jurado, Jason R. Pinner, Katerina Politis, Cathryn Poulton, Theresa Power, Michael Quinn, Sulekha Rajagopalan, Matthew Regan, Jonathan Rodgers, Steuart Rorke, Rani Sachdev, Suzanne Sallevelt, Sarah A. Sandaradura, Maryam Shamassi, Roshan Shamon, Isabella Sherburn, Jennie Slee, Annalisa Solinas, Ella Sugo, Elizabeth Thompson, Sagarika Tripathy, Anand Vasudevan, Melisa Vazquez, Kunal Verma, Mthulisi Viki, Mathew Wallis, Dani L. Webber, Martin Weber, Karen Whale, Meredith Wilson, Lisa Worgan, Sui Yu, George McGillivray, Fiona McKenzie, Jill Lipsett, Nick Manton, Christopher P. Barnett, George McGillivray, Jason Pinner, Fiona McKenzie, Rebecca Morrow, Jill Lipsett, Nick Manton, T. Yee Khong, Lynette Moore, Jan E. Liebelt, Andreas W. Schreiber, Sarah L. King-Smith, Tristan S. E. Hardy, Matilda R. Jackson, Christopher P. Barnett, Hamish S. Scott

**Affiliations:** 1 Department of Genetics and Molecular Pathology, Centre for Cancer Biology, an alliance between SA Pathology and the University of South Australia, Adelaide, South Australia Australia; 2 UniSA Clinical and Health Sciences, University of South Australia, Adelaide, South Australia Australia; 3 Broad Institute of MIT and Harvard, Cambridge, MA USA; 4 Adelaide Medical School, University of Adelaide, Adelaide, South Australia Australia; 5 ACRF Genomics Facility, Centre for Cancer Biology, an alliance between SA Pathology and the University of South Australia, Adelaide, South Australia Australia; 6 Department of Genetics and Molecular Pathology, SA Pathology, Adelaide, South Australia Australia; 7 Division of Genetics and Genomics, Boston Childrens Hospital, Boston, MA USA; 8 Victorian Clinical Genetics Services, Murdoch Children’s Research Institute and Royal Women’s Hospital, Melbourne, Victoria Australia; 9 Centre for Clinical Genetics, Sydney Children’s Hospitals Network – Randwick, Sydney, New South Wales Australia; 10 School of Women’s and Children’s Health, University of NSW, Sydney, New South Wales Australia; 11 Genetic Services of Western Australia, Perth, Western Australia Australia; 12 School of Paediatrics and Child Health, University of Western Australia, Perth, Western Australia Australia; 13 Department of Anatomical Pathology, SA Pathology, Women’s and Children’s Hospital, North Adelaide, South Australia Australia; 14 Paediatric and Reproductive Genetics Unit, South Australian Clinical Genetics Service, Women’s and Children’s Hospital, North Adelaide, South Australia Australia; 15 Repromed, Dulwich, South Australia Australia; 16 School of Biological Sciences, University of Adelaide, Adelaide, South Australia Australia; 17 Australian Genomics, Parkville, Victoria Australia; 18 Center for Genomic Medicine, Massachusetts General Hospital, Boston, MA USA; 19 Program in Bioinformatics and Integrative Genomics, Harvard Medical School, Boston, MA USA; 20 Neuromuscular Division, Department of Neurology, Brigham and Women’s Hospital, Boston, MA USA; 21 Yale School of Medicine, Department of Genetics, New Haven, CT USA; 22 Garvan Institute of Medical Research and UNSW Sydney, Centre for Population Genomics, Sydney, New South Wales Australia; 23 Murdoch Children’s Research Institute, Centre for Population Genomics, Melbourne, Victoria Australia; 24 Boston Childrens Hospital Divisions of Newborn Medicine and Genetics and Genomics, Boston, MA USA; 25 Department of Anatomical Pathology, Perth Children’s Hospital, Nedlands, Western Australia Australia; 26 Department of Clinical Genetics, Children’s Hospital at Westmead, Westmead, New South Wales Australia; 27 Disciplines of Child and Adolescent Health and Genomic Medicine, University of Sydney, Sydney, New South Wales Australia; 28 Murdoch Children’s Research Institute, Parkville, Victoria Australia; 29 Department of Histopathology, The Children’s Hospital at Westmead, Westmead, New South Wales Australia; 30 Department of Clinical Genetics, Liverpool Hospital, Liverpool, New South Wales Australia; 31 Kids Neuroscience Centre, The Children’s Hospital at Westmead, Westmead, New South Wales Australia; 32 Faculty of Medicine and Health, The University of Sydney, Westmead, New South Wales Australia; 33 Department of Anatomical Pathology, Royal Children’s Hospital, Parkville, Victoria Australia; 34 Department of Pathology, Monash Health, Melbourne, Victoria Australia; 35 Department of Anatomical Pathology, Austin Health, Melbourne, Victoria Australia; 36 Clinical Genetics Service, Institute of Precision Medicine and Bioinformatics, Royal Prince Alfred Hospital, Sydney, New South Wales Australia; 37 Functional Neuromics, Children’s Medical Research Institute, Westmead, New South Wales Australia; 38 Robinson Research Institute, The University of Adelaide, Adelaide, South Australia Australia; 39 Department of Anatomical Pathology, Canberra Hospital, Canberra, Australian Capital Territory Australia; 40 ANU Medical School, Canberra, Australian Capital Territory Australia; 41 Neonatal and Pediatric Intensive Care Unit, Department of Pediatrics, Royal Hobart Hospital, Hobart, Tasmania Australia; 42 Department of Pathology, Royal Brisbane and Women’s Hospital, Herston, Brisbane, Queensland Australia; 43 Royal Women’s Hospital, Melbourne, Victoria Australia; 44 Department of Anatomical Pathology, Dorevitch Pathology, Laboratory, Frankston Hospital, Peninsula Health, Victoria Australia; 45 Hunter Genetics, Newcastle, New South Wales Australia; 46 St Vincent’s Clinical School, University of NSW, Sydney, New South Wales Australia; 47 Kinghorn Centre for Clinical Genomics, Garvan Institute of Medical Research, Sydney, New South Wales Australia; 48 Monash Genetics, Monash Health, Melbourne, Victoria Australia; 49 Department of Pediatrics, Monash University, Melbourne, Victoria Australia; 50 Genetics and Molecular Pathology, SA Pathology at Women’s and Children’s Hospital, North Adelaide, South Australia Australia; 51 MotherSafe, Royal Hospital for Women, Sydney, New South Wales Australia; 52 Department of Anatomical Pathology, NSW Health Pathology, Prince of Wales Hospital, Randwick, New South Wales Australia; 53 Department of Medical Genomics, Royal Prince Alfred Hospital, Sydney, New South Wales Australia; 54 Department of Anatomical Pathology, Mater Health Services, Brisbane, Queensland Australia; 55 Genetic Health Queensland, Royal Brisbane and Women’s Hospital, Brisbane, Queensland Australia; 56 Department of Pediatric Genetics, Ankara Bilkent City Hospital Children’s Hospital, Ankara, Turkey; 57 Victorian Institute of Forensic Medicine, Southbank, Victoria Australia; 58 Genetics Service, Hospital del Mar Medical Research Institute, Network Research Centre for Rare Diseases, Barcelona, Spain; 59 Department of Medicine and Life Sciences, Universitat Pompeu Fabra, Barcelona, Spain; 60 Royal Prince Alfred Hospital, Camperdown, New South Wales Australia; 61 Department of Anatomical Pathology, Pathology North Hunter, New Lambton, New South Wales Australia; 62 Centre for Translational Pathology, University of Melbourne, Parkville, Victoria Australia; 63 Genomic Medicine, The Royal Melbourne Hospital, Parkville, Victoria Australia; 64 School of Medicine and Menzies Institute for Medical Research, University of Tasmania, Hobart, Tasmania Australia; 65 Department of Pathology, The Royal Hobart Hospital, Hobart, Tasmania Australia

**Keywords:** Genetic testing, Disease genetics, Medical genomics

Correction to: *Nature Medicine* 10.1038/s41591-022-02142-1. Published online 19 January 2023.

This paper was originally published under a standard Springer Nature license (© Crown, under exclusive licence to Springer Nature America, Inc.). It is now available as an open-access paper under a Creative Commons Attribution 4.0 International license, © Crown. The error has been corrected in the HTML and PDF versions of the article.

