# Supplementary material for: Genomic autopsy to identify underlying causes of pregnancy loss and perinatal death
Source: Nat Med. 2023 Jan 19;29(1):180–9. doi: 10.1038/s41591-022-02142-1 (PMC10333122; doi:10.1038/s41591-022-02142-1)
Supplement: Supplementary file 1 — Reporting Summary [file 41591_2022_2142_MOESM1_ESM.pdf]

nature portfolio

Corresponding author(s): Christopher P Barnett and Hamish S Scott

Last updated by author(s): 11/04/2022

Reporting Summary

Nature Portfolio wishes to improve the reproducibility of the work that we publish. This form provides structure for consistency and transparency in reporting. For further information on Nature Portfolio policies, see our Editorial Policies and the Editorial Policy Checklist.

Statistics

For all statistical analyses, confirm that the following items are present in the figure legend, table legend, main text, or Methods section.

n/a	Confirmed	
☐	☒	The exact sample size (*n*) for each experimental group/condition, given as a discrete number and unit of measurement
☒	☐	A statement on whether measurements were taken from distinct samples or whether the same sample was measured repeatedly
☐	☒	The statistical test(s) used AND whether they are one- or two-sided<br>*Only common tests should be described solely by name; describe more complex techniques in the Methods section.*
☒	☐	A description of all covariates tested
☒	☐	A description of any assumptions or corrections, such as tests of normality and adjustment for multiple comparisons
☐	☒	A full description of the statistical parameters including central tendency (e.g. means) or other basic estimates (e.g. regression coefficient) AND variation (e.g. standard deviation) or associated estimates of uncertainty (e.g. confidence intervals)
☐	☒	For null hypothesis testing, the test statistic (e.g. *F*, *t*, *r*) with confidence intervals, effect sizes, degrees of freedom and *P* value noted<br>*Give P values as exact values whenever suitable.*
☒	☐	For Bayesian analysis, information on the choice of priors and Markov chain Monte Carlo settings
☒	☐	For hierarchical and complex designs, identification of the appropriate level for tests and full reporting of outcomes
☒	☐	Estimates of effect sizes (e.g. Cohen's *d*, Pearson's *r*), indicating how they were calculated

Our web collection on statistics for biologists contains articles on many of the points above.

Software and code

Policy information about availability of computer code

Data collection	A Redcap database (https://www.sciencedirect.com/science/article/pii/S1532046408001226) and VariantGrid (https://github.com/SACGF/variantgrid) were used for data collection
Data analysis	VariantGrid and Seqr were used for genomic analyses. The codes for these online analysis platforms are available on github at https://github.com/SACGF/variantgrid and https://github.com/broadinstitute/seqr, respectively.

For manuscripts utilizing custom algorithms or software that are central to the research but not yet described in published literature, software must be made available to editors and reviewers. We strongly encourage code deposition in a community repository (e.g. GitHub). See the Nature Portfolio guidelines for submitting code & software for further information.

Data

Policy information about availability of data

All manuscripts must include a data availability statement. This statement should provide the following information, where applicable:

- Accession codes, unique identifiers, or web links for publicly available datasets
- A description of any restrictions on data availability
- For clinical datasets or third party data, please ensure that the statement adheres to our policy

All generated and analyzed sequencing data has been deposited at the European Genome-phenome Archive (EGA) which is hosted by the European Bioinformatics Institute (EBI) and the Centre for Genomic Regulation (CRG) under accession number EGAS00001006295.

As the data may contain identifiable information, access to some of the data will be controlled and is available upon reasonable request from the corresponding author, Prof. Hamish S Scott (hamish.scott@sa.gov.au).

Human research participants

Policy information about studies involving human research participants and Sex and Gender in Research.

Reporting on sex and gender	We have used "sex" throughout the manuscript, as we only report biological findings. Sex of the proband and/or their affected sibling was not a determinant for case selection (51.5% is male, 48.5% female), but was considered to enable appropriate genomic variant analysis (e.g. contribution of X-linked genetic variants). Self-reported (parents) or clinically determined (proband and siblings) sex of all study participants were confirmed genomically using Peddy or Somalier, and established sex presented throughout. One proband (PED210) and one affected sibling (PED043) had complete sex reversal as part of their clinical phenotype and an additional seven probands had 'ambiguous genitalia', further complicating a potential sex-based analysis.
Population characteristics	All population characteristics of the cases (deceased fetuses) included in our study are provided in supplementary table 2. The couples experiencing the loss of their pregnancy or newborn, are of childbearing age who have seen a clinical geneticist or genetic counselor. Initially, the study was only recruiting within South Australia, expanding to nation-wide recruitment after the first years. There was no inclusion or exclusion based on other characteristics (such as ethnical background) and we expect the cohort to be representative of the Australian population.
Recruitment	Cases of pregnancy loss (including terminations) and perinatal death with congenital abnormalities, or cases that were clinically completely unexplained were recruited into our study after standard-of-care testing in a public healthcare setting did not provide them with a likely genetic diagnosis. As described in the online methods: While these cases were sequentially referred, they do not represent the complete spectrum of pregnancy loss and perinatal death as only patients within the public healthcare system were recruited, and only cases with consent were approved into the study. To evaluate the percentage of cases compared to the fetal and perinatal loss population, we have reviewed one year of clinical data. In that year (2017) 234 local cases of fetal loss and perinatal death were discussed during weekly MDT meetings at the Women's and Children's hospital in Adelaide (with 97 families the main referral site for our study, and the only one where we have insight in these data). Of these 234 cases, 80 were assessed as being potentially eligible for our study, 31 were referred and 18 were recruited. From this review, we conclude that clinically biased ascertainment is unlikely because of a thorough assessment of cases at an MDT meeting, more families are recommended for referral than are actually referred (from 2017 about 40% who could potentially have been referred were referred), and once referred, over half were recruited (60% in 2017). There is no clinical selection bias except potentially obstetrician-gynaecologists not referring, which would be random.
Ethics oversight	This study was performed as part of the NHMRC and GHFM-MRFF funded Genomic Autopsy Study, and was approved by the Human Ethics Committee of the Women's and Children's Health Network, South Australia, Australia (HREC/15/WCHN/35) and the Melbourne Health Human Research Ethics Committee as part of the Australian Genomics Health Alliance protocol: HREC/16/MH/251. Informed consent for genomic analysis and participation in study protocols was obtained from parents, and all research was conducted in accordance with the Declaration of Helsinki.

Note that full information on the approval of the study protocol must also be provided in the manuscript.

Field-specific reporting

Please select the one below that is the best fit for your research. If you are not sure, read the appropriate sections before making your selection.

☒ Life sciences ☐ Behavioural & social sciences ☐ Ecological, evolutionary & environmental sciences

For a reference copy of the document with all sections, see nature.com/documents/nr-reporting-summary-flat.pdf

Life sciences study design

All studies must disclose on these points even when the disclosure is negative.

Sample size	The first 200 consecutively referred cases (families) were included in the study.
Data exclusions	No data were excluded from analysis
Replication	No replication of exome or genome sequencing was required. However, sequencing variants detected in research variants were confirmed (by Sanger sequencing and/or droplet digital PCR), for clinically accredited genomic analysis this wasn't required if the variant call reached QC requirements.
Randomization	There was no difference in the study protocol, whereby every participant followed the same workflow of testing until a (likely) cause is identified, so randomisation and blinding are not applicable to study design

Blinding | There was no difference in the study protocol, whereby every participant followed the same workflow of testing until a (likely) cause is identified, so randomisation and blinding are not applicable to study design

Reporting for specific materials, systems and methods

We require information from authors about some types of materials, experimental systems and methods used in many studies. Here, indicate whether each material, system or method listed is relevant to your study. If you are not sure if a list item applies to your research, read the appropriate section before selecting a response.

Materials & experimental systems

n/a	Involved in the study
☒	☐ Antibodies
☒	☐ Eukaryotic cell lines
☒	☐ Palaeontology and archaeology
☒	☐ Animals and other organisms
☒	☐ Clinical data
☒	☐ Dual use research of concern

Methods

n/a	Involved in the study
☒	☐ ChIP-seq
☒	☐ Flow cytometry
☒	☐ MRI-based neuroimaging
